# Stochastic dynamical analysis for the complex infectious disease model driven by multisource noises

**DOI:** 10.1371/journal.pone.0296183

**Published:** 2024-01-04

**Authors:** Liqiong Jian, Xinyu Bai, Shaojuan Ma

**Affiliations:** 1 The Blood Center of Ningxia Hui Autonomous Region, Yinchuan, China; 2 School of Mathematics and Information Science, North Minzu University, Yinchuan, China; Southwest University, CHINA

## Abstract

This paper mainly studies the dynamical behavior of the infectious disease model affected by white noise and Lévy noise. First, a stochastic model of infectious disease with secondary vaccination affected by noises is established. Besides, the existence and uniqueness of the global positive solution for the stochastic model are proved based on stochastic differential equations and Lyapunov function, then the asymptotic behavior of the disease-free equilibrium point is studied. Moreover, the sufficient conditions for the extinction of the disease are obtained and the analysis showed that different noise intensity could affect the extinction of infectious disease on different degree. Finally, the theoretical results are verified by numerical simulation and some suggestions have been put forward on how to prevent the spread of diseases are presented.

## 1 Introduction

Infectious diseases can be transmitted between people, animals and goods which always threaten human survival and take great challenges to whole world [1–3]. In recent decades, a large number of mathematical models for infectious diseases have been built and studied widely to realize the infectious disease. The basic mathematical model representing the dynamical behavior of the three main populations which include the susceptible(S(t)), the infected(I(t)) and the recovered(R(t)), was firstly proposed in 1927 by Kermack and McKendricks [4] called SIR model. Two metapopulation SIR models from individual and population perspectives were proposed in reference [5], which studied the significant influence of contact-dependent infection and migration on epidemic propagation. Khyar et al. [6] considered the multi strain SEIR epidemic model with general incidence rate and gave the equilibrium point stability theorem of different strains. Reference [7] analyzed modified SLIR model with nonlinear incidence and equilibriums of the proposed model are both globally asymptotically stable. Gumel et al. [8] proposed extended models of COVID-19 in which the stability of the equilibrium point and parameter estimation was studied.

As we all know, the accurate modeling can more effectively explore the mechanism of the infectious disease. Therefore, the main factors reflected the practical infectious diseases must be considered, such as delay-time, vaccination and random disturbance. Vaccination [9–12] has always been one of the effective measures to control infectious diseases. Xing et al. [13] studied a recurrent nonautonomous SVIR epidemic model with vaccination, who proved the existence and uniqueness of globally attractive near periodic solutions for the model. A deterministic SVIRS epidemic model with Holling type II incidence rate and vaccination was investigated in reference [14], which explicitly discussed the local stability of the disease equilibrium and the existence of Hopf bifurcation. In order to enhance the immune effect and increase the probability of antibody production, most vaccines adopt a vaccination program of two or more doses. Gabrick et al. [15] propose a SEIR model with two doses of vaccine administration, and analyze that administering two doses of vaccine can significantly reduce the number of infections. Omar et al. [16] generated fractional order model based on the secondary vaccination and analyzed various vaccination strategies. Reference [17] established the SIRS model by introducing vaccination passes and made predictions based on real-world parameter values.

In real life, infectious diseases are inevitably influenced by various random factors during the transmission process, so considering the influence of random factors in infectious disease models will be more practical. In addition, many studies shown that environmental fluctuations also have a huge impact on the development of epidemics with vaccination. Therefore, stochastic differential equation model [18–20] became a more appropriate method for modeling epidemic diseases. A stochastic cholera model with saturation recovery rate is discussed in the reference [21], then the optimal control is added and studied to provide a theoretical basis for the prevention and control of cholera. Zhang et al. [22] established a stochastic *SVIR* model with general incidence rate, who obtained the sufficient conditions of the extinction and persistence for the model affected by white noise. Reference [23] built a SIVS epidemic model with white noise and gave sufficient conditions of the existence for the periodic solutions. It can be found in reference [24] that the random threshold of the outcome for the stochastic SIS model with vaccination can be determined in case the white noises are small. The SVIR model with white noise was proposed in reference [25], which showed that that environmental white noise is helpful for controlling the disease. Reference [26] further shown that the disease gradually disappeared due to the influence of environmental white noise in the stochastic COVID-19 model. Wang et al. [27] studied the influence of vaccination rates, vaccine effectiveness and immune loss rates on infectious disease in a stochastic mathematical model with vaccination. We can conclude from the above references that appropriate white noise intensity can accelerate the extinction of diseases under some conditions.

However, some sudden environmental impacts such as earthquakes, floods, large scale human activities, etc. also affect the infectious diseases in reality which cannot be described by white noise. Therefore, researchers began to use non Gaussian Lévy noise to depict these sudden environmental phenomena in nature [28, 29]. Reference [30] presents a class of the stochastic time-delayed SEIRS epidemic model incorporating both public health education driven by Lévy noise and how to prevent and control diseases. Sabbar et al. [31] established a stochastic COVID model under the influence of quadratic Lévy noise and quadratic jump-diffusion has no impact on the threshold value, but it remarkably influences the dynamics of the infection and may worsen the pandemic situation. The stochastic SIRS epidemic model with white noise and Lévy noise was discussed by Mu et al. [32], who verified that Lévy noise can further inhibit the outbreak of disease. Fan [33] proposed a stochastic SIV epidemic model with non-linear incidence rate, who found that the Lévy noise intensity and the vaccination have a great impact on the transmission dynamics of the disease. Reference [34] introduced white noise together with Lévy noise into the stochastic SIVS model and proposed random threshold affected by noise intensity which completely determined the development of epidemic disease. In addition, a hybrid switching SVIR epidemic model with Lévy noise was studied in reference [35] and the sufficient conditions for the existence of positive recursion for the solutions were obtained. Jaouad et al. [36] established a stochastic infectious disease model with isolation strategy for COVID-19 and studied the stochastic dynamical properties of the random solutions around the equilibrium point. Based on the above references, although various mathematical models have been extensively studied and applied, it should be noted that the complex modeling of secondary vaccination for infectious diseases is still not enough and the propagation mechanism of the corresponding complex models is not clear. In addition, the research on discontinuous random disturbances in such vaccination models should be further explored. Therefore, building upon the foundation established by previous works, this study seeks to explore the dynamical properties of the stochastic model with secondary vaccination, which is mainly driven by discontinuous noise, namely Lévy noise. In particular, we will discuss the effects of discontinuous stochastic interference and its intensity on the average extinction of diseases.

The present work will be organized as follows. In the second section, we establish the disease model with secondary vaccination and multisource noises. The existence and uniqueness of positive solutions proposing model is proved in the third section. In the fourth section, asymptotic behavior at the disease-free equilibrium point is studied and analyzed. We give the random threshold of disease extinction for the proposed model in the fifth section. In the last section, the theoretical results are verified by numerical simulation.

## 2 Stochastic model

In this paper, in order to be more consistent with the early development of infectious disease and obtain the required results, we present the following assumptions:

(1)The recovered population has immunity and will not be infected again.
(2)Most people who receive the second vaccination will have immunity and will not be infected again.
(3)Vaccinated people will transfer to exposure after being infected.
(4)Assuming that the interval between two vaccinations is short, the first vaccinated person will not be infected temporarily.

**Fig 1:**
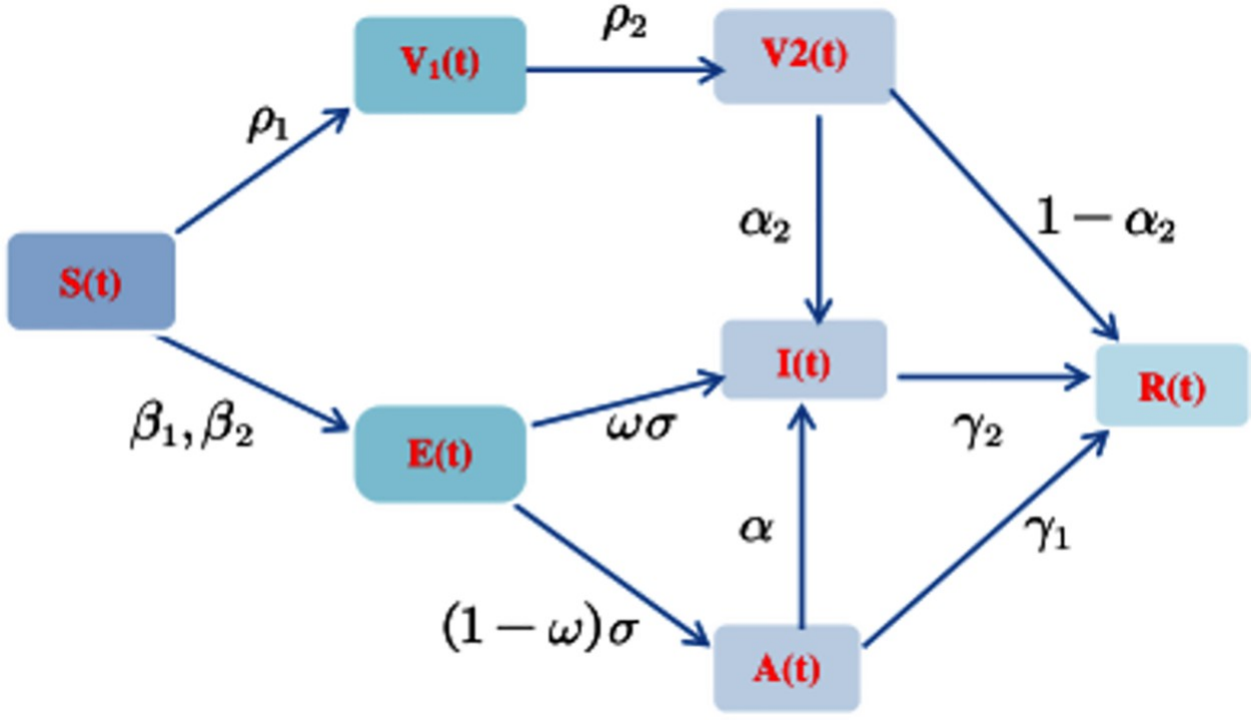
The relationship diagram between different compartments in the infectious disease model.

We divide the population into seven compartments based on the above assumptions as shown in Fig 1. That is susceptible person *S*(*t*), exposed person *E*(*t*), first vaccinator *V*_1_(*t*), second vaccinator *V*_2_(*t*), asymptomatic infected person *A*(*t*), symptomatic infected person *I*(*t*) and recovering person *R*(*t*). By introducing white noise, the model is built to reflect the more real state of disease transmission. Among them, white noise represents small disturbances in the environment, such as temperature changes, climate and other impacts. The established stochastic model is as follows [37]:

$$\begin{array}{c} \left\{ \begin{array}{cc} & \text{d}S=[\Lambda -\beta_{1}SA-\beta_{2}SI-(\mu_{1}+\rho_{1})S]\text{d}t+\sigma_{1}S\text{d}B_{1}\left(t\right),\\ & \text{d}E=[\beta_{1}SA+\beta_{2}SI+\alpha_{2}\beta_{2}V_{2}I-(\sigma +\mu_{1})E]\text{d}t+\sigma_{2}E\text{d}B_{2}\left(t\right),\\ & \text{d}V_{1}=[\rho_{1}S-(\rho_{2}+\mu_{1})V_{1}]\text{d}t+\sigma_{3}V_{1}\text{d}B_{3}\left(t\right),\\ & \text{d}V_{2}=[\rho_{2}V_{1}-\mu_{1}V_{2}-\alpha_{2}\beta_{2}V_{2}I-(1-\alpha_{2})V_{2}]\text{d}t+\sigma_{4}V_{2}\text{d}B_{4}\left(t\right),\\ & \text{d}A=[\left(1-\omega \right)\sigma E-(\alpha +\gamma_{1}+\mu_{1}+\mu_{2})A]\text{d}t+\sigma_{5}A\text{d}B_{5}\left(t\right),\\ & \text{d}I=[\omega \sigma E+\alpha A-(\gamma_{2}+\mu_{1}+\mu_{2})I]\text{d}t+\sigma_{6}I\text{d}B_{6}\left(t\right),\\ & \text{d}R=[(1-\alpha_{2})V_{2}+\gamma_{1}A+\gamma_{2}I-\mu_{1}R]\text{d}t+\sigma_{7}R\text{d}B_{7}\left(t\right), \end{array}\right. \end{array}$$ (1)

where Λ is the constant migration rate of the susceptible population, *β*_1_ is the transmission rate of asymptomatic infected persons, *β*_2_ is the transmission rate of symptomatic infected persons, *ρ*_1_ is the first vaccination rate, *ρ*_2_ is the second vaccination rate, *σ* is the infection rate of the exposed to the infected, *ω* is the proportion of infections in symptomatic patients, *α* is the ratio from asymptomatic infected people to symptomatic infected people, *α*_2_(0 < *α*_2_ < 1) is the infection rate of secondary vaccinators, so 1 − *α*_2_ is the effectiveness of the vaccine. *γ*_1_ and *γ*_2_ respectively represent the recovery rate of asymptomatic and symptomatic infected persons. *μ*_1_ and *μ*_2_ represent the natural mortality rate and disease-related mortality rate respectively. All parameters are positive. *B*_*i*_(*t*), *i* = 1, 2, 3, ⋯, 7 denotes the independent standard Brownian motion defined on a complete probability space (Ω, *F*, *P*) with filtering {*F*_*t*_}_*t*>0_, *σ*_*i*_ ≥ 0 is the intensity of *B*_*i*_(*t*), *i* = 1, 2, 3, ⋯, 7.

When sudden phenomena occur in nature, the impact of measures taken by people during the epidemic and government intervention on infectious diseases is described by Lévy noise in this article. Therefore, a class of improved models driven by both white noise and Lévy noise are as follows:

$$\begin{array}{c} \left\{ \begin{array}{cc} \text{d}S= & [\Lambda -\beta_{1}SA-\beta_{2}SI-(\mu_{1}+\rho_{1})S]\text{d}t+\sigma_{1}S\text{d}B_{1}\left(t\right)\\ & +\int_{Y}D_{1}\left(u\right)S\left(t\right)\tilde{N}\left(\text{d}t,\text{d}u\right),\\ \text{d}E= & [\beta_{1}SA+\beta_{2}SI+\alpha_{2}\beta_{2}V_{2}I-(\sigma +\mu_{1})E]\text{d}t+\sigma_{2}E\text{d}B_{2}\left(t\right)\\ & +\int_{Y}D_{2}\left(u\right)E\left(t\right)\tilde{N}\left(\text{d}t,\text{d}u\right),\\ \text{d}V_{1}= & [\rho_{1}S-(\rho_{2}+\mu_{1})V_{1}]\text{d}t+\sigma_{3}V_{1}\text{d}B_{3}\left(t\right)\\ & +\int_{Y}D_{3}\left(u\right)V_{1}\left(t\right)\tilde{N}\left(\text{d}t,\text{d}u\right),\\ \text{d}V_{2}= & [\rho_{2}V_{1}-\mu_{1}V_{2}-\alpha_{2}\beta_{2}V_{2}I-(1-\alpha_{2})V_{2}]\text{d}t+\sigma_{4}V_{2}\text{d}B_{4}\left(t\right)\\ & +\int_{Y}D_{4}\left(u\right)V_{2}\left(t\right)\tilde{N}\left(\text{d}t,\text{d}u\right),\\ \text{d}A= & [\left(1-\omega \right)\sigma E-(\alpha +\gamma_{1}+\mu_{1}+\mu_{2})A]\text{d}t+\sigma_{5}A\text{d}B_{5}\left(t\right)\\ & +\int_{Y}D_{5}\left(u\right)A\left(t\right)\tilde{N}\left(\text{d}t,\text{d}u\right),\\ \text{d}I= & [\omega \sigma E+\alpha A-(\gamma_{2}+\mu_{1}+\mu_{2})I]\text{d}t+\sigma_{6}I\text{d}B_{6}\left(t\right)\\ & +\int_{Y}D_{6}\left(u\right)I\left(t\right)\tilde{N}\left(\text{d}t,\text{d}u\right),\\ \text{d}R= & [(1-\alpha_{2})V_{2}+\gamma_{1}A+\gamma_{2}I-\mu_{1}R]\text{d}t+\sigma_{7}R\text{d}B_{7}\left(t\right)\\ & +\int_{Y}D_{7}\left(u\right)R\left(t\right)\tilde{N}\left(\text{d}t,\text{d}u\right), \end{array}\right. \end{array}$$ (2)

where $\tilde{N}\left(\text{d}t,\text{d}u\right)=N\left(\text{d}t,\text{d}u\right)-\lambda \left(\text{d}u\right)\text{d}t$ is Poisson compensation measure, *N*(d*t*, d*u*) is Poisson random measure, λ(d*u*)d*t* is smooth compensation. λ is a feature measure defined on a measurable subset of *Y* ⊂ [0, ∞) and satisfies λ(*Y*) < ∞. In addition, *N*(d*t*, d*u*) and *B*_*i*_(*t*) are independent, *D*_*i*_(*u*) > −1(*i* = 1, 2, 3, ⋯, 7) represents the jump diffusion coefficient.

For *σ*_*i*_(*i* = 1, 2, 3, ⋯, 7) and *D*_*i*_(*u*)(*i* = 1, 2, 3, ⋯, 7) of Eq (2), we make the following assumptions:

(A) *D*_*i*_(*u*) is bounded and $|\frac{\Lambda }{\mu_{1}}D_{i}\left(u\right)|\leq \delta <1\left(i=1,2,3,\cdots ,7\right)$, where *u* ∈ *Y*.
(B) Suppose *ϵ* > 1 exists make
  $$\begin{array}{c} \mu_{1}-\frac{\epsilon -1}{2}\sigma^{2}-\frac{d}{\epsilon }>0, \end{array}$$
  where
  $$\begin{array}{c} \sigma^{2}=\sigma_{1}^{2}\vee \sigma_{2}^{2}\vee \sigma_{3}^{2}\vee \sigma_{4}^{2}\vee \sigma_{5}^{2}\vee \sigma_{6}^{2}\vee \sigma_{7}^{2}, \end{array}$$
  $$\begin{array}{c} d=\int_{Y}[{\left(1+D_{1}\left(u\right)\vee D_{2}\left(u\right)\vee D_{3}\left(u\right)\vee D_{4}\left(u\right)\vee D_{5}\left(u\right)\vee D_{6}\left(u\right)\vee D_{7}\left(u\right)\right)}^{\epsilon }\\ -1-D_{1}\left(u\right)\wedge D_{2}\left(u\right)\wedge D_{3}\left(u\right)\wedge D_{4}\left(u\right)\wedge D_{5}\left(u\right)\wedge D_{6}\left(u\right)\wedge D_{7}\left(u\right)]\lambda \text{d}u. \end{array}$$
(C) There is a normal number *D* that satisfies
  $$\begin{array}{c} \int_{Y}{\left(\text{ln}\left(1+D_{i}\left(u\right)\right)\right)}^{2}\lambda \left(\text{d}u\right)<D,\;i=1,2,3,\cdots ,7. \end{array}$$

It should be pointed out that Eq (2) is an idealized model. From a real perspective, Eq (2) can approximately describe the spread of diseases in areas with sudden random population changes. In the following content, we mainly focus on how the discontinuous environmental shocks described by Lévy jumps affect the dynamic behavior of Eq (2), especially the threshold for disease extinction.

## 3 Existence and uniqueness of global positive solution

In order to study the dynamics of the infectious disease model, we first concern whether the solution of Eq (2) is global and unique. Here, we give the following theorem. Since the first six formulas of Eq (2) which do not contain *R*(*t*), this section only considers the subsystem composed of the first six formulas of Eq (2), i.e Eq (3):

$$\begin{array}{c} \left\{ \begin{array}{cc} \text{d}S= & [\Lambda -\beta_{1}SA-\beta_{2}SI-(\mu_{1}+\rho_{1})S]\text{d}t+\sigma_{1}S\text{d}B_{1}\left(t\right)\\ & +\int_{Y}D_{1}\left(u\right)S\left(t\right)\tilde{N}\left(\text{d}t,\text{d}u\right),\\ \text{d}E= & [\beta_{1}SA+\beta_{2}SI+\alpha_{2}\beta_{2}V_{2}I-(\sigma +\mu_{1})E]\text{d}t+\sigma_{2}E\text{d}B_{2}\left(t\right)\\ & +\int_{Y}D_{2}\left(u\right)E\left(t\right)\tilde{N}\left(\text{d}t,\text{d}u\right),\\ \text{d}V_{1}= & [\rho_{1}S-(\rho_{2}+\mu_{1})V_{1}]\text{d}t+\sigma_{3}V_{1}\text{d}B_{3}\left(t\right)\\ & +\int_{Y}D_{3}\left(u\right)V_{1}\left(t\right)\tilde{N}\left(\text{d}t,\text{d}u\right),\\ \text{d}V_{2}= & [\rho_{2}V_{1}-\mu_{1}V_{2}-\alpha_{2}\beta_{2}V_{2}I-(1-\alpha_{2})V_{2}]\text{d}t+\sigma_{4}V_{2}\text{d}B_{4}\left(t\right)\\ & +\int_{Y}D_{4}\left(u\right)V_{2}\left(t\right)\tilde{N}\left(\text{d}t,\text{d}u\right),\\ \text{d}A= & [\left(1-\omega \right)\sigma E-(\alpha +\gamma_{1}+\mu_{1}+\mu_{2})A]\text{d}t+\sigma_{5}A\text{d}B_{5}\left(t\right)\\ & +\int_{Y}D_{5}\left(u\right)A\left(t\right)\tilde{N}\left(\text{d}t,\text{d}u\right),\\ \text{d}I= & [\omega \sigma E+\alpha A-(\gamma_{2}+\mu_{1}+\mu_{2})I]\text{d}t+\sigma_{6}I\text{d}B_{6}\left(t\right)\\ & +\int_{Y}D_{6}\left(u\right)I\left(t\right)\tilde{N}\left(\text{d}t,\text{d}u\right). \end{array}\right. \end{array}$$ (3)

**Theorem 3.1** *Let Assumption (A) hold, for given initial value* (*S*(0), *E*(0), *V*_1_(0), *V*_2_(0), *A*(0), *I*(0)), Eq (3) *has a unique positive solution* (*S*(*t*), *E*(*t*), *V*_1_(*t*), *V*_2_(*t*), *A*(*t*), *I*(*t*)) *at t* ≥ 0, *and this solution will stay in* $R_{+}^{6}$ *with probability 1. So for all t* ≥ 0, *solution* $\left(S\left(t\right),E\left(t\right),V_{1}\left(t\right),V_{2}\left(t\right),A\left(t\right),I\left(t\right)\right)\in R_{+}^{6}$ *a.s*..

Proof: Since the coefficients of the equation are locally Lipschitz continuous, for given initial value (*S*(0), *E*(0), $V_{1}\left(0\right),V_{2}\left(0\right),A\left(0\right),I\left(0\right))\in R_{+}^{6}$, there is a unique local solution (*S*(*t*), *E*(*t*), *V*_1_(*t*), *V*_2_(*t*), *A*(*t*), *I*(*t*)), *t* ∈ [0, *τ*_*e*_), where *τ*_*e*_ stands for explosion time. To prove that the local solution is the global solution, we need to prove *τ*_*e*_ = ∞ a.s.. To do that, we have to make *k*_0_ > 0 and sufficiently large, so that *S*(*t*), *E*(*t*), *V*_1_(*t*), *V*_2_(*t*), *A*(*t*), *I*(*t*) is in the interval $\left[\frac{1}{k_{0}},k_{0}\right]$. For each integer *k* ≥ *k*_0_, we define the stopping time

$$\begin{array}{cc} \tau_{k}= & \text{inf}\{t\in [0,\tau_{e}):S\left(t\right)\notin (\frac{1}{k},k)\;\text{or}\;E\left(t\right)\notin (\frac{1}{k},k)\;\text{or}\;V_{1}\left(t\right)\notin (\frac{1}{k},k)\\ & \text{or}\;V_{2}\left(t\right)\notin (\frac{1}{k},k)\;\text{or}\;A\left(t\right)\notin (\frac{1}{k},k)\;\text{or}\;I\left(t\right)\notin (\frac{1}{k},k)\}. \end{array}$$

In this section, we set inf ∅ = ∞ (∅ denotes the empty set). It is easy to get *τ*_*k*_ is increasing as *k* → ∞. Set *τ*_∞_ = lim_*k*→∞_ *τ*_*k*_ which implies *τ*_∞_ < *τ*_*e*_ a.s.. If the hypothesis *τ*_∞_ = ∞ is true, then *τ*_*e*_ = ∞ a.s.. For all *t* ≥ 0. This means

$$\begin{array}{c} \{S\left(t\right),E\left(t\right),V_{1}\left(t\right),V_{2}\left(t\right),A\left(t\right),I\left(t\right)\}\in R_{+}^{6}\;a.s.. \end{array}$$

Use proof by contradiction, assume *τ*_*e*_ = ∞ a.s.. And then there are constants *T* > 0 and *ε* ∈ (0, 1) which make

$$\begin{array}{c} P\{\tau_{\infty }\leq T\}\geq \varepsilon . \end{array}$$

Hence there is an integer *k*_1_ > *k*_0_ such that

$$\begin{array}{c} P\{\tau_{k}\leq T\}\geq \varepsilon ,\;\forall k>k_{1}. \end{array}$$ (4)

Define a *C*^2^–function $V:R_{+}^{6}\to R_{+}$, as follows

$$\begin{array}{cc} & V(S,E,V_{1},V_{2},A,I)\\ = & \left(S-a-a\;\text{ln}\;\frac{S}{a}\right)+\left(E-1-\text{ln}\;E\right)+(V_{1}-1-\text{ln}\;V_{1})+\\ & (V_{2}-1-\text{ln}\;V_{2})+\left(A-1-\text{ln}\;A\right)+\left(I-1-\text{ln}\;I\right), \end{array}$$ (5)

the non-negativity of Eq (5) can be obtained from

$$\begin{array}{c} u-1-\text{ln}\;u\geq 0,\;\forall u>0. \end{array}$$

Using Itô formula, we can get

$$\begin{array}{cc} & \text{d}V(S,E,V_{1},V_{2},A,I)\\ = & LV(S,E,V_{1},V_{2},A,I,R)\text{d}t+\sigma_{1}\left(S-1\right)\text{d}B_{1}\left(t\right)+\sigma_{2}\left(E-1\right)\text{d}B_{2}\left(t\right)\\ & +\sigma_{3}(V_{1}-1)\text{d}B_{3}\left(t\right)+\sigma_{4}(V_{2}-1)\text{d}B_{4}\left(t\right)+\sigma_{5}\left(A-1\right)\text{d}B_{5}\left(t\right)\\ & +\sigma_{6}\left(I-1\right)\text{d}B_{6}\left(t\right)+a\int_{Y}\left[D_{1}\left(u\right)S-\text{ln}\left(1+D_{1}\left(u\right)S\right)\right]\tilde{N}\left(\text{d}t,\text{d}u\right)\\ & +\int_{Y}\left[D_{2}\left(u\right)E-\text{ln}\left(1+D_{2}\left(u\right)E\right)\right]\tilde{N}\left(\text{d}t,\text{d}u\right)\\ & +\int_{Y}\left[D_{3}\left(u\right)V_{1}-\text{ln}\left(1+D_{3}\left(u\right)V_{1}\right)\right]\tilde{N}\left(\text{d}t,\text{d}u\right)\\ & +\int_{Y}\left[D_{4}\left(u\right)V_{2}-\text{ln}\left(1+D_{4}\left(u\right)V_{2}\right)\right]\tilde{N}\left(\text{d}t,\text{d}u\right)\\ & +\int_{Y}\left[D_{5}\left(u\right)A-\text{ln}\left(1+D_{5}\left(u\right)A\right)\right]\tilde{N}\left(\text{d}t,\text{d}u\right)\\ & +\int_{Y}\left[D_{6}\left(u\right)I-\text{ln}\left(1+D_{6}\left(u\right)I\right)\right]\tilde{N}\left(\text{d}t,\text{d}u\right), \end{array}$$ (6)

where $LV:R_{+}^{6}\to R_{+}$ is defined by

$$\begin{array}{cc} & LV(S,E,V_{1},V_{2},A,I)\\ = & \left(1-\frac{a}{S})[\Lambda -\beta_{1}SA-\beta_{2}SI-(\mu_{1}+\rho_{1})S\right]\\ & +(1-\frac{1}{E})[\beta_{1}SA+\beta_{2}SI+\alpha_{2}\beta_{2}V_{2}I-(\sigma +\mu_{1})E]\\ & +(1-\frac{1}{V_{1}})[\rho_{1}S-(\rho_{2}+\mu_{1})V_{1}]\\ & +(1-\frac{1}{V_{2}})[\rho_{2}V_{1}-\mu_{1}V_{2}-\alpha_{2}\beta_{2}V_{2}I-(1-\alpha_{2})V_{2}]\\ & +(1-\frac{1}{A})[\left(1-\omega \right)\sigma E-(\alpha +\gamma_{1}+\mu_{1}+\mu_{2})A]\\ & +(1-\frac{1}{I})[\omega \sigma E+\alpha A-(\gamma_{2}+\mu_{1}+\mu_{2})I]\\ & +\frac{1}{2}(\sigma_{1}^{2}+\sigma_{2}^{2}+\sigma_{3}^{2}+\sigma_{4}^{2}+\sigma_{5}^{2}+\sigma_{6}^{2})+a\int_{Y}\left[D_{1}\left(u\right)S-\text{ln}\left(1+D_{1}\left(u\right)S\right)\right]\lambda \left(\text{d}u\right)\\ & +\int_{Y}\left[D_{2}\left(u\right)E-\text{ln}\left(1+D_{2}\left(u\right)E\right)\right]\lambda \left(\text{d}u\right)+\int_{Y}\left[D_{3}\left(u\right)V_{1}-\text{ln}\left(1+D_{3}\left(u\right)V_{1}\right)\right]\lambda \left(\text{d}u\right)\\ & +\int_{Y}\left[D_{4}\left(u\right)V_{2}-\text{ln}\left(1+D_{4}\left(u\right)V_{2}\right)\right]\lambda \left(\text{d}u\right)+\int_{Y}\left[D_{5}\left(u\right)A-\text{ln}\left(1+D_{5}\left(u\right)A\right)\right]\lambda \left(\text{d}u\right)\\ & +\int_{Y}\left[D_{6}\left(u\right)I-\text{ln}\left(1+D_{6}\left(u\right)I\right)\right]\lambda \left(\text{d}u\right)\\ ⩽ & \Lambda +a\left(\rho_{1}+\mu_{1}\right)+\sigma +\rho_{2}+(1-\alpha_{2})+\alpha +\gamma_{1}+\gamma_{2}+5\mu_{1}+2\mu_{2}+a\left(\beta_{1}A+\beta_{2}I\right)\\ & -\left(\gamma_{1}+\mu_{1}+\mu_{2}\right)A-\left(\gamma_{2}+\mu_{1}+\mu_{2}\right)I+\frac{1}{2}(\sigma_{1}^{2}+\sigma_{2}^{2}+\sigma_{3}^{2}+\sigma_{4}^{2}+\sigma_{5}^{2}+\sigma_{6}^{2})\\ & +a\int_{Y}\left[D_{1}\left(u\right)S-\text{ln}\left(1+D_{1}\left(u\right)S\right)\right]\lambda \left(\text{d}u\right)+\int_{Y}\left[D_{2}\left(u\right)E-\text{ln}\left(1+D_{2}\left(u\right)E\right)\right]\lambda \left(\text{d}u\right)\\ & +\int_{Y}\left[D_{3}\left(u\right)V_{1}-\text{ln}\left(1+D_{3}\left(u\right)V_{1}\right)\right]\lambda \left(\text{d}u\right)+\int_{Y}\left[D_{4}\left(u\right)V_{2}-\text{ln}\left(1+D_{4}\left(u\right)V_{2}\right)\right]\lambda \left(\text{d}u\right)\\ & +\int_{Y}\left[D_{5}\left(u\right)A-\text{ln}\left(1+D_{5}\left(u\right)A\right)\right]\lambda \left(\text{d}u\right)+\int_{Y}\left[D_{6}\left(u\right)I-\text{ln}\left(1+D_{6}\left(u\right)I\right)\right]\lambda \left(\text{d}u\right), \end{array}$$

we let

$$\begin{array}{c} a=\frac{\gamma_{1}+\mu_{1}+\mu_{2}}{\beta_{1}}=\frac{\gamma_{2}+\mu_{1}+\mu_{2}}{\beta_{2}}, \end{array}$$

there are

$$\begin{array}{cc} & LV(S,E,V_{1},V_{2},A,I)\\ ⩽ & \Lambda +a\left(\rho_{1}+\mu_{1}\right)+\sigma +\rho_{2}+(1-\alpha_{2})+\alpha +\gamma_{1}+\gamma_{2}+5\mu_{1}+2\mu_{2}\\ & +\frac{1}{2}(\sigma_{1}^{2}+\sigma_{2}^{2}+\sigma_{3}^{2}+\sigma_{4}^{2}+\sigma_{5}^{2}+\sigma_{6}^{2})+a\int_{Y}\left[D_{1}\left(u\right)S-\text{ln}\left(1+D_{1}\left(u\right)S\right)\right]\lambda \left(\text{d}u\right)\\ & +\int_{Y}\left[D_{2}\left(u\right)E-\text{ln}\left(1+D_{2}\left(u\right)E\right)\right]\lambda \left(\text{d}u\right)+\int_{Y}\left[D_{3}\left(u\right)V_{1}-\text{ln}\left(1+D_{3}\left(u\right)V_{1}\right)\right]\lambda \left(\text{d}u\right)\\ & +\int_{Y}\left[D_{4}\left(u\right)V_{2}-\text{ln}\left(1+D_{4}\left(u\right)V_{2}\right)\right]\lambda \left(\text{d}u\right)+\int_{Y}\left[D_{5}\left(u\right)A-\text{ln}\left(1+D_{5}\left(u\right)A\right)\right]\lambda \left(\text{d}u\right)\\ & +\int_{Y}\left[D_{6}\left(u\right)I-\text{ln}\left(1+D_{6}\left(u\right)I\right)\right]\lambda \left(\text{d}u\right) \end{array}$$

$$\begin{array}{cc} ⩽ & \Lambda +a\left(\rho_{1}+\mu_{1}\right)+\sigma +\rho_{2}+(1-\alpha_{2})+\alpha +\gamma_{1}+\gamma_{2}+5\mu_{1}+2\mu_{2}\\ & +\frac{1}{2}(\sigma_{1}^{2}+\sigma_{2}^{2}+\sigma_{3}^{2}+\sigma_{4}^{2}+\sigma_{5}^{2}+\sigma_{6}^{2})+a\int_{Y}H_{1}\lambda \left(\text{d}u\right)+\int_{Y}H_{2}\lambda \left(\text{d}u\right)\\ & +\int_{Y}H_{3}\lambda \left(\text{d}u\right)+\int_{Y}H_{4}\lambda \left(\text{d}u\right)+\int_{Y}H_{5}\lambda \left(\text{d}u\right)+\int_{Y}H_{6}\lambda \left(\text{d}u\right), \end{array}$$

where

$$\begin{array}{cc} H_{1} & =D_{1}\left(u\right)S-\text{ln}\left(1+D_{1}\left(u\right)S\right),\;H_{2}=D_{2}\left(u\right)E-\text{ln}\left(1+D_{2}\left(u\right)E\right),\\ H_{3} & =D_{3}\left(u\right)V_{1}-\text{ln}\left(1+D_{3}\left(u\right)V_{1}\right),\;H_{4}=D_{4}\left(u\right)V_{2}-\text{ln}\left(1+D_{4}\left(u\right)V_{2}\right),\\ H_{5} & =D_{5}\left(u\right)A-\text{ln}\left(1+D_{5}\left(u\right)A\right),\;H_{6}=D_{6}\left(u\right)I-\text{ln}\left(1+D_{6}\left(u\right)I\right). \end{array}$$

Using assumption (A) and Taylor’s formula

$$\begin{array}{c} \begin{array}{cc} H_{1} & =\frac{D_{1}^{2}\left(u\right)S^{2}}{2{\left(1-\theta_{1}D_{1}\left(u\right)S\right)}^{2}}\leq \frac{\delta^{2}}{2{\left(1-\delta \right)}^{2}},\;H_{2}=\frac{D_{2}^{2}\left(u\right)E^{2}}{2{\left(1-\theta_{2}D_{2}\left(u\right)S\right)}^{2}}\leq \frac{\delta^{2}}{2{\left(1-\delta \right)}^{2}},\\ H_{3} & =\frac{D_{3}^{2}\left(u\right)V_{1}^{2}}{2{\left(1-\theta_{3}D_{3}\left(u\right)V_{1}\right)}^{2}}\leq \frac{\delta^{2}}{2{\left(1-\delta \right)}^{2}},\;H_{4}=\frac{D_{4}^{2}\left(u\right)V_{2}^{2}}{2{\left(1-\theta_{4}D_{4}\left(u\right)V_{2}\right)}^{2}}\leq \frac{\delta^{2}}{2{\left(1-\delta \right)}^{2}},\\ H_{5} & =\frac{D_{5}^{2}\left(u\right)A^{2}}{2{\left(1-\theta_{5}D_{5}\left(u\right)A\right)}^{2}}\leq \frac{\delta^{2}}{2{\left(1-\delta \right)}^{2}},\;H_{6}=\frac{D_{6}^{2}\left(u\right)I^{2}}{2{\left(1-\theta_{6}D_{6}\left(u\right)I\right)}^{2}}\leq \frac{\delta^{2}}{2{\left(1-\delta \right)}^{2}}, \end{array} \end{array}$$

where *θ*_*i*_(*i* = 1, ⋯, 6) is any value in (0, 1).

So we can get

$$\begin{array}{cc} & LV(S,E,V_{1},V_{2},A,I)\\ ⩽ & \Lambda +a\left(\rho_{1}+\mu_{1}\right)+\sigma +\rho_{2}+(1-\alpha_{2})+\alpha +\gamma_{1}+\gamma_{2}+5\mu_{1}+2\mu_{2}\\ & +\frac{1}{2}(\sigma_{1}^{2}+\sigma_{2}^{2}+\sigma_{3}^{2}+\sigma_{4}^{2}+\sigma_{5}^{2}+\sigma_{6}^{2})+\left(a+5\right)\int_{Y}\frac{\delta^{2}}{2{\left(1-\delta \right)}^{2}}\lambda \left(\text{d}u\right)\\ ⩽ & \Lambda +a\left(\rho_{1}+\mu_{1}\right)+\sigma +\rho_{2}+(1-\alpha_{2})+\alpha +\gamma_{1}+\gamma_{2}+5\mu_{1}+2\mu_{2}\\ & +\frac{1}{2}(\sigma_{1}^{2}+\sigma_{2}^{2}+\sigma_{3}^{2}+\sigma_{4}^{2}+\sigma_{5}^{2}+\sigma_{6}^{2})+\left(a+5\right)\frac{\delta^{2}}{2{\left(1-\delta \right)}^{2}}\lambda \left(Y\right)\\ : & =K, \end{array}$$

where *K*(*K* ∈ *N*^+^) is a positive constant, which isn’t rely on *S*, *E*, *V*_1_, *V*_2_, *A*, *I* and *t*.

So there is

$$\begin{array}{cc} & \text{d}V(S,E,V_{1},V_{2},A,I)\\ ⩽ & K\text{d}t+\sigma_{1}\left(S-a\right)\text{d}B_{1}\left(t\right)+\sigma_{2}\left(E-1\right)\text{d}B_{2}\left(t\right)+\sigma_{3}(V_{1}-1)\text{d}B_{3}\left(t\right)\\ & +\sigma_{4}(V_{2}-1)\text{d}B_{4}\left(t\right)+\sigma_{5}\left(A-1\right)\text{d}B_{5}\left(t\right)+\sigma_{6}\left(I-1\right)\text{d}B_{6}\left(t\right)\\ & +a\int_{Y}\left[D_{1}\left(u\right)S-\text{ln}\left(1+D_{1}\left(u\right)S\right)\right]\tilde{N}\left(\text{d}t,\text{d}u\right)\\ & +\int_{Y}\left[D_{2}\left(u\right)E-\text{ln}\left(1+D_{2}\left(u\right)E\right)\right]\tilde{N}\left(\text{d}t,\text{d}u\right)\\ & +\int_{Y}\left[D_{3}\left(u\right)V_{1}-\text{ln}\left(1+D_{3}\left(u\right)V_{1}\right)\right]\tilde{N}\left(\text{d}t,\text{d}u\right)\\ & +\int_{Y}\left[D_{4}\left(u\right)V_{2}-\text{ln}\left(1+D_{4}\left(u\right)V_{2}\right)\right]\tilde{N}\left(\text{d}t,\text{d}u\right)\\ & +\int_{Y}\left[D_{5}\left(u\right)A-\text{ln}\left(1+D_{5}\left(u\right)A\right)\right]\tilde{N}\left(\text{d}t,\text{d}u\right)\\ & +\int_{Y}\left[D_{6}\left(u\right)I-\text{ln}\left(1+D_{6}\left(u\right)I\right)\right]\tilde{N}\left(\text{d}t,\text{d}u\right). \end{array}$$ (7)

Integrating both sides of Eq (7) from 0 to *τ*_*k*_ ∧ *T* = min{*τ*_*k*_, *T*}, then take the expectation, we can get

$$\begin{array}{c} \begin{array}{cc} & EV(S(\tau_{k}\wedge T),E(\tau_{k}\wedge T),V_{1}(\tau_{k}\wedge T),V_{2}(\tau_{k}\wedge T),A(\tau_{k}\wedge T),I(\tau_{k}\wedge T))\\ ⩽ & V(S\left(0\right),E\left(0\right),V_{1}\left(0\right),V_{2}\left(0\right),A\left(0\right),I\left(0\right))+KE(\tau_{k}\wedge T)\\ ⩽ & V(S\left(0\right),E\left(0\right),V_{1}\left(0\right),V_{2}\left(0\right),A\left(0\right),I\left(0\right))+KT. \end{array} \end{array}$$ (8)

Set Ω_*k*_ = {*τ*_*k*_ ≤ *T*} when *k* ≥ *k*_1_, we obtain *P*{Ω_*k*_} ≥ *ε* by Eq (4). Now notice that for every *ω* ∈ Ω_*k*_, there is at least one of *S*(*τ*_*k*_, *ω*), *E*(*τ*_*k*_, *ω*), *V*_1_(*τ*_*k*_, *ω*), *V*_2_(*τ*_*k*_, *ω*), *A*(*τ*_*k*_, *ω*), *I*(*τ*_*k*_, *ω*) equals to *k* or $\frac{1}{k}$.

Therefore

$$\begin{array}{c} V\{S(\tau_{k}\wedge T),E(\tau_{k}\wedge T),V_{1}(\tau_{k}\wedge T),V_{2}(\tau_{k}\wedge T),A(\tau_{k}\wedge T),I(\tau_{k}\wedge T)\} \end{array}$$

is no less than

$$\begin{array}{c} k-1-\text{ln}\;k\;\text{or}\;\frac{1}{k}-1-\text{ln}\frac{1}{k}\;\text{or}\;k-a-a\;\text{ln}\frac{k}{a}\;\text{or}\;\frac{1}{k}-a-a\;\text{ln}\frac{1}{ak}, \end{array}$$

thus

$$\begin{array}{c} \begin{array}{cc} & \;V\{S(\tau_{k}\wedge T),E(\tau_{k}\wedge T),V_{1}(\tau_{k}\wedge T),V_{2}(\tau_{k}\wedge T),A(\tau_{k}\wedge T),I(\tau_{k}\wedge T)\}\\ & \geq \text{min}\{k-1-\text{ln}\;k,\;\;\frac{1}{k}-1-\text{ln}\frac{1}{k},\;k-a-a\;\text{ln}\frac{k}{a},\;\frac{1}{k}-a-a\;\text{ln}\frac{1}{ak}\}. \end{array} \end{array}$$ (9)

Substituting Eq (8) into Eq (9), we have

$$\begin{array}{c} \begin{array}{cc} & V(S\left(0\right),E\left(0\right),V_{1}\left(0\right),V_{2}\left(0\right),A\left(0\right),I\left(0\right))+KT\\ ⩾ & E[1_{\Omega_{k}\left(\omega \right)}\forall V\{S(\tau_{k}\wedge T),E(\tau_{k}\wedge T),V_{1}(\tau_{k}\wedge T),V_{2}(\tau_{k}\wedge T),A(\tau_{k}\wedge T),I(\tau_{k}\wedge T)\}]\\ ⩾ & \varepsilon \;\text{min}\{k-1-\text{ln}\;k,\;\;\frac{1}{k}-1-\text{ln}\frac{1}{k},\;k-a-a\;\text{ln}\frac{k}{a},\;\frac{1}{k}-a-a\;\text{ln}\frac{1}{ak}\}, \end{array} \end{array}$$ (10)

where $1_{\Omega_{k}(\omega )}$ denotes the indicator function of Ω_*k*_. Letting *k* → ∞, then we have

$$\begin{array}{c} \infty >V(S\left(0\right),E\left(0\right),V_{1}\left(0\right),V_{2}\left(0\right),A\left(0\right),I\left(0\right))+KT=\infty , \end{array}$$ (11)

Eq (11) is a contradiction. Therefore we have *τ*_∞_ = ∞, i.e the proof is completed.

From Theorem 3.1, it can be seen that regardless of the noise intensity *D*_*i*_ and *σ*_*i*_, the stochastic model almost always has a unique global positive solution for any given initial value.

## 4 Asymptotic behavior of disease-free equilibrium point

The disease-free equilibrium point of the deterministic form of Eq (1) can be obtained

$$\begin{array}{c} P_{0}=\left(S^{*},0,V_{1}^{*},V_{2}^{*},0,0,R^{*}\right), \end{array}$$

where

$$\begin{array}{c} \begin{array}{cc} S^{*} & =\frac{\Lambda }{\mu_{1}+\rho_{1}},\;V_{1}^{*}=\frac{\Lambda \rho_{1}}{\left(\mu_{1}+\rho_{1})(\mu_{1}+\rho_{2}\right)},\\ V_{2}^{*} & =\frac{\Lambda \rho_{1}\rho_{2}}{\left(\mu_{1}+\rho_{1})(\mu_{1}+\rho_{2})(\mu_{1}+1-\alpha_{2}\right)},\\ R^{*} & =\frac{\Lambda \rho_{1}\rho_{2}(1-\alpha_{2})}{\mu_{1}(\mu_{1}+\rho_{1})(\mu_{1}+\rho_{2})(\mu_{1}+1-\alpha_{2})}. \end{array} \end{array}$$

Based on the disease-free equilibrium point and its expression obtained from the above equation, we can then discuss the asymptotic behavior of the system’s solution in the disease-free equilibrium point of the corresponding deterministic system, which reflects whether the disease is extinct to a certain extent.

**Theorem 4.1** *For any given initial value* (*S*(0), *E*(0), *V*_1_(0), *V*_2_(0), *A*(0), *I*(0), *R*(0)) $\in R_{+}^{7}$, *then the solution of the* Eq (2) *satisfies the following property*

$$\begin{array}{c} \begin{array}{c} \underset{t\to \infty }{\text{lim}}\frac{1}{t}E\int_{0}^{t}[{\left(S\left(s\right)-S^{*}\right)}^{2}+E{\left(s\right)}^{2}+{\left(V_{1}\left(s\right)-V_{1}^{*}\right)}^{2}+{\left(V_{2}\left(s\right)-V_{2}^{*}\right)}^{2}\\ \;+A{\left(s\right)}^{2}+I{\left(s\right)}^{2}+{\left(R\left(s\right)-R^{*}\right)}^{2}]\text{d}s\leq \frac{M}{q}, \end{array} \end{array}$$

*where*

$$\begin{array}{c} \begin{array}{cc} M= & \frac{2\Lambda \rho_{2}\left(1-\alpha_{2}\right)}{5\mu_{1}\rho_{1}\left(\mu_{1}+1-\alpha_{2}\right)\left(\mu_{1}+\rho_{2}\right)}+\frac{1}{5}\left[\left(1-\alpha_{2}\right)V_{2}^{*}-\mu_{1}R^{*}\right]+\frac{1}{4}c_{1}^{2}\rho_{1}^{2}\\ & +\left[2\sigma_{1}^{2}+6\int_{Y}D_{1}^{2}\left(u\right)\lambda \left(\text{d}u\right)\right]{\left(S^{*}\right)}^{2}+\left[2\sigma_{3}^{2}+6\int_{Y}D_{3}^{2}\left(u\right)\lambda \left(\text{d}u\right)\right]{\left(V_{1}^{*}\right)}^{2}\\ & +\left[2\sigma_{4}^{2}+6\int_{Y}D_{4}^{2}\left(u\right)\lambda \left(\text{d}u\right)\right]{\left(V_{2}^{*}\right)}^{2},\\ q= & \text{min}\{q_{1},q_{2},q_{3},q_{4},q_{5},q_{6}\},\\ q_{1}= & 2\mu_{1}+1-\sigma_{1}^{2}-6\int_{Y}D_{1}^{2}\left(u\right)\lambda \left(\text{d}u\right),\;q_{2}=2\mu_{1}-\sigma_{2}^{2}-3\int_{Y}D_{2}^{2}\left(u\right)\lambda \left(\text{d}u\right),\\ q_{3}= & 2\mu_{1}-\sigma_{3}^{2}-6\int_{Y}D_{3}^{2}\left(u\right)\lambda \left(\text{d}u\right),\;q_{4}=2\mu_{1}+1-\alpha_{2}-\sigma_{4}^{2}-6\int_{Y}D_{4}^{2}\left(u\right)\lambda \left(\text{d}u\right),\\ q_{5}= & 2\mu_{1}+2\mu_{2}+2\gamma_{1}-\sigma_{5}^{2}-3\int_{Y}D_{5}^{2}\left(u\right)\lambda \left(\text{d}u\right),\\ q_{6}= & 2\mu_{1}+2\mu_{2}+2\gamma_{2}-\sigma_{6}^{2}-6\int_{Y}D_{6}^{2}\left(u\right)\lambda \left(\text{d}u\right). \end{array} \end{array}$$

Proof: Let

$$\begin{array}{c} \begin{array}{cc} x(t) & =S\left(t\right)-S^{*},\;y\left(t\right)=E\left(t\right),\;z\left(t\right)=V_{1}\left(t\right)-V_{1}^{*},\;k\left(t\right)=V_{2}\left(t\right)-V_{2}^{*},\\ m(t) & =A\left(t\right),\;n\left(t\right)=I\left(t\right),\;l\left(t\right)=R\left(t\right)-R^{*}. \end{array} \end{array}$$

Eq (2) is rewritten as

$$\begin{array}{c} \left\{ \begin{array}{cc} \text{d}x(t)= & [-\beta_{1}\left(x\left(t\right)+S^{*}\right)m\left(t\right)-\beta_{2}\left(x\left(t\right)+S^{*}\right)n\left(t\right)-(\mu_{1}+\rho_{1})x\left(t\right)]\text{d}t\\ & +\sigma_{1}\left(x\left(t\right)+S^{*}\right)\text{d}B_{1}\left(t\right)+\int_{Y}D_{1}\left(u\right)\left(x\left(t\right)+S^{*}\right)\tilde{N}\left(\text{d}t,\text{d}u\right),\\ \text{d}y(t)= & [\beta_{1}\left(x\left(t\right)+S^{*}\right)m\left(t\right)+\beta_{2}\left(x\left(t\right)+S^{*}\right)n\left(t\right)+\alpha_{2}\beta_{2}\left(k\left(t\right)+V_{2}^{*}\right)n\left(t\right)\\ & -(\sigma +\mu_{1})y\left(t\right)]\text{d}t+\sigma_{2}y\left(t\right)\text{d}B_{2}\left(t\right)+\int_{Y}D_{2}\left(u\right)y\left(t\right)\tilde{N}\left(\text{d}t,\text{d}u\right),\\ \text{d}z(t)= & [\rho_{1}\left(x\left(t\right)+S^{*}\right)-(\rho_{2}+\mu_{1})z\left(t\right)-\frac{\Lambda }{\mu_{1}+\rho_{1}}]\text{d}t\\ & +\sigma_{3}\left(z\left(t\right)+V_{1}^{*}\right)\text{d}B_{3}\left(t\right)+\int_{Y}D_{3}\left(u\right)\left(z\left(t\right)+V_{1}^{*}\right)\tilde{N}\left(\text{d}t,\text{d}u\right), \end{array}\right. \end{array}$$

$$\begin{array}{c} \left\{ \begin{array}{cc} \text{d}k(t)= & [\rho_{2}\left(z\left(t\right)+V_{1}^{*}\right)-\alpha_{2}\beta_{2}\left(k\left(t\right)+V_{2}^{*}\right)n\left(t\right)-(\mu_{1}+1-\alpha_{2})k\left(t\right)\\ & +\frac{\Lambda \rho_{1}\rho_{2}}{\left(\mu_{1}+\rho_{1}\right)\left(\mu_{1}+\rho_{2}\right)}]\text{d}t+\sigma_{4}\left(k\left(t\right)+V_{2}^{*}\right)\text{d}B_{4}\left(t\right)\\ & +\int_{Y}D_{4}\left(u\right)\left(k\left(t\right)+V_{2}^{*}\right)\tilde{N}\left(\text{d}t,\text{d}u\right),\\ \text{d}m(t)= & [\left(1-\omega \right)\sigma y\left(t\right)-(\alpha +\gamma_{1}+\mu_{1}+\mu_{2})m\left(t\right)]\text{d}t\\ & +\sigma_{5}m\left(t\right)\text{d}B_{5}\left(t\right)+\int_{Y}D_{5}\left(u\right)m\left(t\right)\tilde{N}\left(\text{d}t,\text{d}u\right),\\ \text{d}n(t)= & [\omega \sigma y\left(t\right)+\alpha m\left(t\right)-(\gamma_{2}+\mu_{1}+\mu_{2})n\left(t\right)]\text{d}t\\ & +\sigma_{6}n\left(t\right)\text{d}B_{6}\left(t\right)+\int_{Y}D_{6}\left(u\right)n\left(t\right)\tilde{N}\left(\text{d}t,\text{d}u\right),\\ \text{d}l(t)= & [(1-\alpha_{2})\left(k\left(t\right)+V_{2}^{*}\right)+\gamma_{1}m\left(t\right)+\gamma_{2}n\left(t\right)-\mu_{1}\left(l\left(t\right)+R^{*}\right)]\text{d}t\\ & +\sigma_{7}\left(l\left(t\right)+R^{*}\right)\text{d}B_{7}\left(t\right)+\int_{Y}D_{7}\left(u\right)\left(l\left(t\right)+R^{*}\right)\tilde{N}\left(\text{d}t,\text{d}u\right). \end{array}\right. \end{array}$$

Define a *C*^2^–function

$$\begin{array}{c} \begin{array}{cc} & W(x,y,z,k,m,n,l)\\ = & {\left(x+y+z+k+m+n\right)}^{2}+c_{1}y+c_{2}z+c_{3}k+c_{4}m+c_{5}n+\frac{1}{5}l, \end{array} \end{array}$$

where *c*_*i*_(*i* = 1, ⋯, 5) are the normal numbers to be determined.

Using Itô formula, we have

$$\begin{array}{c} \begin{array}{cc} & \text{d}W\\ = & LW\text{d}t+2\left(x+y+z+k+m+n\right)[\sigma_{1}\left(x\left(t\right)+S^{*}\right)\text{d}B_{1}\left(t\right)+\sigma_{2}y\left(t\right)\text{d}B_{2}\left(t\right)\\ & +\sigma_{3}\left(z\left(t\right)+V_{1}^{*}\right)\text{d}B_{3}\left(t\right)+\sigma_{4}\left(k\left(t\right)+V_{2}^{*}\right)\text{d}B_{4}\left(t\right)+\sigma_{5}m\left(t\right)\text{d}B_{5}\left(t\right)\\ & +\sigma_{6}n\left(t\right)\text{d}B_{6}\left(t\right)]+c_{1}\sigma_{2}y\left(t\right)\text{d}B_{2}\left(t\right)+c_{2}\sigma_{3}\left(z\left(t\right)+V_{1}^{*}\right)\text{d}B_{3}\left(t\right)\\ & +c_{3}\sigma_{4}\left(k\left(t\right)+V_{2}^{*}\right)\text{d}B_{4}\left(t\right)+c_{4}\sigma_{5}m\left(t\right)\text{d}B_{5}\left(t\right)+c_{5}\sigma_{6}n\left(t\right)\text{d}B_{6}\left(t\right)\\ & +\frac{1}{5}\sigma_{7}\left(l\left(t\right)+R^{*}\right)\text{d}B_{7}\left(t\right)+2\int_{Y}\left(x+y+z+k+m+n\right)[D_{1}\left(u\right)\left(x\left(t\right)+S^{*}\right)\\ & +D_{2}\left(u\right)y\left(t\right)+D_{3}\left(u\right)\left(z\left(t\right)+V_{1}^{*}\right)+D_{4}\left(u\right)\left(k\left(t\right)+V_{2}^{*}\right)+D_{5}\left(u\right)m\left(t\right)\\ & +D_{6}\left(u\right)n\left(t\right)]\tilde{N}\left(\text{d}t,\text{d}u\right)+\int_{Y}[D_{1}\left(u\right)\left(x\left(t\right)+S^{*}\right)+D_{2}\left(u\right)y\left(t\right)\\ & +D_{3}\left(u\right)\left(z\left(t\right)+V_{1}^{*}\right)+D_{4}\left(u\right)\left(k\left(t\right)+V_{2}^{*}\right)\\ & +D_{5}\left(u\right)m\left(t\right)+D_{6}{\left(u\right)n\left(t\right)]}^{2}\tilde{N}\left(\text{d}t,\text{d}u\right)\\ & +c_{1}\int_{Y}D_{2}\left(u\right)y\left(t\right)\tilde{N}\left(\text{d}t,\text{d}u\right)+c_{2}\int_{Y}D_{3}\left(u\right)\left(z\left(t\right)+V_{1}^{*}\right)\tilde{N}\left(\text{d}t,\text{d}u\right)\\ & +c_{3}\int_{Y}D_{4}\left(u\right)\left(k\left(t\right)+V_{2}^{*}\right)\tilde{N}\left(\text{d}t,\text{d}u\right)+c_{4}\int_{Y}D_{5}\left(u\right)m\left(t\right)\tilde{N}\left(\text{d}t,\text{d}u\right)\\ & +c_{5}\int_{Y}D_{6}\left(u\right)n\left(t\right)\tilde{N}\left(\text{d}t,\text{d}u\right)+\frac{1}{5}\int_{Y}D_{7}\left(u\right)\left(l\left(t\right)+R^{*}\right)\tilde{N}\left(\text{d}t,\text{d}u\right), \end{array} \end{array}$$ (12)

where

$$\begin{array}{c} \begin{array}{cc} LW & =2\left(x+y+z+k+m+n\right)[-\mu_{1}x\left(t\right)-\mu_{1}y\left(t\right)-\mu_{1}z\left(t\right)\\ & -\left(\mu_{1}+1-\alpha_{2}\right)k\left(t\right)-\left(\gamma_{1}+\mu_{1}+\mu_{2}\right)m\left(t\right)-\left(\gamma_{2}+\mu_{1}+\mu_{2}\right)n\left(t\right)]\\ & +c_{1}[\beta_{1}\left(x\left(t\right)+S^{*}\right)m\left(t\right)+\beta_{2}\left(x\left(t\right)+S^{*}\right)n\left(t\right)+\alpha_{2}\beta_{2}\left(k\left(t\right)+V_{2}^{*}\right)n\left(t\right)\\ & -(\sigma +\mu_{1})y\left(t\right)]+c_{2}\left[\rho_{1}\left(x\left(t\right)+S^{*}\right)-(\rho_{2}+\mu_{1})z\left(t\right)-\frac{\Lambda }{\mu_{1}+\rho_{1}}\right]\\ & +c_{3}[\rho_{2}\left(z\left(t\right)+V_{1}^{*}\right)-\alpha_{2}\beta_{2}\left(k\left(t\right)+V_{2}^{*}\right)n\left(t\right)-(\mu_{1}+1-\alpha_{2})k\left(t\right)\\ & +\frac{\Lambda \rho_{1}\rho_{2}}{\left(\mu_{1}+\rho_{1}\right)\left(\mu_{1}+\rho_{2}\right)}]+c_{4}\left[\left(1-\omega \right)\sigma y\left(t\right)-(\alpha +\gamma_{1}+\mu_{1}+\mu_{2})m\left(t\right)\right]\\ & +c_{5}\left[\omega \sigma y\left(t\right)+\alpha m\left(t\right)-(\gamma_{2}+\mu_{1}+\mu_{2})n\left(t\right)\right]\\ & +\frac{1}{5}\left[(1-\alpha_{2})\left(k\left(t\right)+V_{2}^{*}\right)+\gamma_{1}m\left(t\right)+\gamma_{2}n\left(t\right)-\mu_{1}\left(l\left(t\right)+R^{*}\right)\right]\\ & +\sigma_{1}^{2}{\left(x\left(t\right)+S^{*}\right)}^{2}+\sigma_{2}^{2}y{\left(t\right)}^{2}+\sigma_{3}^{2}{\left(z\left(t\right)+V_{1}^{*}\right)}^{2}+\sigma_{4}^{2}{\left(k\left(t\right)+V_{2}^{*}\right)}^{2}\\ & +\sigma_{5}^{2}m{\left(t\right)}^{2}+\sigma_{6}^{2}n{\left(t\right)}^{2}+\int_{Y}[D_{1}\left(u\right)\left(x\left(t\right)+S^{*}\right)+D_{2}\left(u\right)y\left(t\right)\\ & +D_{3}\left(u\right)\left(z\left(t\right)+V_{1}^{*}\right)+D_{4}\left(u\right)\left(k\left(t\right)+V_{2}^{*}\right)+D_{5}\left(u\right)m\left(t\right)+D_{6}{\left(u\right)n\left(t\right)]}^{2}\lambda \left(\text{d}u\right). \end{array} \end{array}$$

At this time, we make

$$\begin{array}{c} \begin{array}{cc} c_{1} & =\frac{2\gamma_{1}+4\mu_{1}+2\mu_{2}}{\beta_{1}}=\frac{2\gamma_{2}+4\mu_{1}+2\mu_{2}}{\beta_{2}},\\ c_{2} & =\frac{\left(1-\alpha_{2}\right)\rho_{2}}{5\left(\mu_{1}+1-\alpha_{2}\right)\left(\mu_{1}+\rho_{2}\right)},\;\;\;\;c_{3}=\frac{1-\alpha_{2}}{5(\mu_{1}+1-\alpha_{2})},\\ c_{4} & =\frac{\omega }{\omega \left(\alpha +\gamma_{1}+\mu_{1}+\mu_{2}\right)+\alpha \left(1-\omega \right)}[\frac{2\Lambda (\gamma_{1}+2\mu_{1}+\mu_{2})}{\mu_{1}+\rho_{1}}\\ & +\frac{2\alpha \left(\gamma_{1}+2\mu_{1}+\mu_{2}\right)\left(\sigma +\mu_{1}\right)}{\beta_{1}\sigma \omega }+\frac{1}{5}\gamma_{1}],\\ c_{5} & =\frac{2\left(\gamma_{1}+2\mu_{1}+\mu_{2}\right)\left(\sigma +\mu_{1}\right)}{\beta_{1}\sigma \omega }\left[1-\frac{\alpha \sigma \omega (1-\omega )}{\omega \left(\alpha +\gamma_{1}+\mu_{1}+\mu_{2}\right)+\alpha \left(1-\omega \right)}\right]\\ & \;-\frac{\sigma \omega (1-\omega )}{\omega \left(\alpha +\gamma_{1}+\mu_{1}+\mu_{2}\right)+\alpha \left(1-\omega \right)}\left[\frac{2\Lambda (\gamma_{1}+2\mu_{1}+\mu_{2})}{\mu_{1}+\rho_{1}}+\frac{1}{5}\gamma_{1}\right], \end{array} \end{array}$$

where *c*_*i*_ > 0, *i* = 1, ⋯, 5, then we use 2*ab* ≤ *a*^2^ + *b*^2^ and (*a* + *b* + *c*)^2^ ≤ 3*a*^2^ + 3*b*^2^ + 3*c*^2^ to get

$$\begin{array}{c} \begin{array}{cc} LW\leq & -\left(2\mu_{1}+1-\sigma_{1}^{2}-6\int_{Y}D_{1}^{2}\left(u\right)\lambda \left(\text{d}u\right)\right)x^{2}-\left(2\mu_{1}-\sigma_{2}^{2}-3\int_{Y}D_{2}^{2}\left(u\right)\lambda \left(\text{d}u\right)\right)y^{2}\\ & -\left(2\mu_{1}-\sigma_{3}^{2}-6\int_{Y}D_{3}^{2}\left(u\right)\lambda \left(\text{d}u\right)\right)z^{2}-\left(2\mu_{1}+1-\alpha_{2}-\sigma_{4}^{2}-6\int_{Y}D_{4}^{2}\left(u\right)\lambda \left(\text{d}u\right)\right)k^{2}\\ & -\left(2\mu_{1}+2\mu_{2}+2\gamma_{1}-\sigma_{5}^{2}-3\int_{Y}D_{5}^{2}\left(u\right)\lambda \left(\text{d}u\right)\right)m^{2}\\ & -\left(2\mu_{1}+2\mu_{2}+2\gamma_{2}-\sigma_{6}^{2}-6\int_{Y}D_{6}^{2}\left(u\right)\lambda \left(\text{d}u\right)\right)n^{2}\\ & +\frac{2\Lambda \rho_{2}\left(1-\alpha_{2}\right)}{5\mu_{1}\rho_{1}\left(\mu_{1}+1-\alpha_{2}\right)\left(\mu_{1}+\rho_{2}\right)}+\frac{1}{5}\left[\left(1-\alpha_{2}\right)V_{2}^{*}-\mu_{1}R^{*}\right]+\frac{1}{4}c_{1}^{2}\rho_{1}^{2}\\ & +\left[2\sigma_{1}^{2}+6\int_{Y}D_{1}^{2}\left(u\right)\lambda \left(\text{d}u\right)\right]{\left(S^{*}\right)}^{2}+\left[2\sigma_{3}^{2}+6\int_{Y}D_{3}^{2}\left(u\right)\lambda \left(\text{d}u\right)\right]{\left(V_{1}^{*}\right)}^{2}\\ & +\left[2\sigma_{4}^{2}+6\int_{Y}D_{4}^{2}\left(u\right)\lambda \left(\text{d}u\right)\right]{\left(V_{2}^{*}\right)}^{2}, \end{array} \end{array}$$

therefore

$$\begin{array}{c} \begin{array}{c} LW\leq -q_{1}x^{2}-q_{2}y^{2}-q_{3}z^{2}-q_{4}k^{2}-q_{5}m^{2}-q_{6}n^{2}+M, \end{array} \end{array}$$ (13)

where

$$\begin{array}{c} \begin{array}{cc} q_{1} & =2\mu_{1}+1-\sigma_{1}^{2}-6\int_{Y}D_{1}^{2}\left(u\right)\lambda \left(\text{d}u\right),\;q_{2}=2\mu_{1}-\sigma_{2}^{2}-3\int_{Y}D_{2}^{2}\left(u\right)\lambda \left(\text{d}u\right),\\ q_{3} & =2\mu_{1}-\sigma_{3}^{2}-6\int_{Y}D_{3}^{2}\left(u\right)\lambda \left(\text{d}u\right),\;q_{4}=2\mu_{1}+1-\alpha_{2}-\sigma_{4}^{2}-6\int_{Y}D_{4}^{2}\left(u\right)\lambda \left(\text{d}u\right),\\ q_{5} & =2\mu_{1}+2\mu_{2}+2\gamma_{1}-\sigma_{5}^{2}-3\int_{Y}D_{5}^{2}\left(u\right)\lambda \left(\text{d}u\right),\\ q_{6} & =2\mu_{1}+2\mu_{2}+2\gamma_{2}-\sigma_{6}^{2}-6\int_{Y}D_{6}^{2}\left(u\right)\lambda \left(\text{d}u\right),\\ M & =\frac{2\Lambda \rho_{2}\left(1-\alpha_{2}\right)}{5\mu_{1}\rho_{1}\left(\mu_{1}+1-\alpha_{2}\right)\left(\mu_{1}+\rho_{2}\right)}+\frac{1}{5}\left[\left(1-\alpha_{2}\right)V_{2}^{*}-\mu_{1}R^{*}\right]+\frac{1}{4}c_{1}^{2}\rho_{1}^{2}\\ & +\left[2\sigma_{1}^{2}+6\int_{Y}D_{1}^{2}\left(u\right)\lambda \left(\text{d}u\right)\right]{\left(S^{*}\right)}^{2}+\left[2\sigma_{3}^{2}+6\int_{Y}D_{3}^{2}\left(u\right)\lambda \left(\text{d}u\right)\right]{\left(V_{1}^{*}\right)}^{2}\\ & +\left[2\sigma_{4}^{2}+6\int_{Y}D_{4}^{2}\left(u\right)\lambda \left(\text{d}u\right)\right]{\left(V_{2}^{*}\right)}^{2}. \end{array} \end{array}$$

Let integrate both sides of Eq (12) from 0 to *τ*_*k*_ ∧ *T* = min{*τ*_*k*_, *T*}, then take the expectation, we can obtain

$$\begin{array}{c} \begin{array}{cc} EW & \left(x,y,z,k,m,n,l\right)\\ = & W\left(x\left(0\right),y\left(0\right),z\left(0\right),k\left(0\right),m\left(0\right),n\left(0\right),l\left(0\right)\right)+E\int_{0}^{t}LWds. \end{array} \end{array}$$ (14)

Substituting Eq (13) into Eq (14) to get

$$\begin{array}{c} \begin{array}{cc} 0 & \leq EW(x,y,z,k,m,n,l)\\ & \leq W(x(0),y(0),z(0),k(0),m(0),n(0),l(0))\\ & +E\int_{0}^{t}\left(-q_{1}x^{2}-q_{2}y^{2}-q_{3}z^{2}-q_{4}k^{2}-q_{5}m^{2}-q_{6}n^{2}\right)ds+Mt. \end{array} \end{array}$$ (15)

We conclude that

$$\begin{array}{c} \begin{array}{c} \underset{t\to \infty }{\text{lim}}\frac{1}{t}E\int_{0}^{t}[{\left(S\left(s\right)-S^{*}\right)}^{2}+E{\left(s\right)}^{2}+{\left(V_{1}\left(s\right)-V_{1}^{*}\right)}^{2}+{\left(V_{2}\left(s\right)-V_{2}^{*}\right)}^{2}\\ \;+A{\left(s\right)}^{2}+I{\left(s\right)}^{2}+{\left(R\left(s\right)-R^{*}\right)}^{2}]\text{d}s\leq \frac{M}{q}, \end{array} \end{array}$$

where

$$\begin{array}{c} \begin{array}{c} q=\text{min}\{q_{1},q_{2},q_{3},q_{4},q_{5},q_{6}\}. \end{array} \end{array}$$

hence, this theorem has proven that the disease-free equilibrium point is globally asymptotically stable.

According to the above theorem, if *P*_0_ is globally asymptotically stable, the disease will disappear for a period of time.

## 5 Extinction of disease

In this section, we shall discuss the condition of extinction of the diseases. To this end, we first provide the following relevant lemma and its proof.

**Lemma 5.1** *For any initial value*

$$\begin{array}{c} \left(S\left(0\right),E\left(0\right),V_{1}\left(0\right),V_{2}\left(0\right),A\left(0\right),I\left(0\right),R\left(0\right)\right)\in R_{+}^{7}, \end{array}$$

Eq (2) *always has a unique global positive solution*

$$\begin{array}{c} \left(S\left(t\right),E\left(t\right),V_{1}\left(t\right),V_{2}\left(t\right),A\left(t\right),I\left(t\right),R\left(t\right)\right)\in R_{+}^{7}\;at\;t\geq 0, \end{array}$$

*it has the following properties*

$$\begin{array}{c} \begin{array}{cc} \underset{t\to \infty }{\text{lim}}\frac{S(t)}{t} & =0,\underset{t\to \infty }{\text{lim}}\frac{E(t)}{t}=0,\underset{t\to \infty }{\text{lim}}\frac{V_{1}\left(t\right)}{t}=0,\underset{t\to \infty }{\text{lim}}\frac{V_{2}\left(t\right)}{t}=0,\\ \underset{t\to \infty }{\text{lim}}\frac{A(t)}{t} & =0,\underset{t\to \infty }{\text{lim}}\frac{I(t)}{t}=0,\underset{t\to \infty }{\text{lim}}\frac{R(t)}{t}=0, \end{array} \end{array}$$ (16)

*and*

$$\begin{array}{c} \begin{array}{cc} & \underset{t\to \infty }{\text{lim}}\frac{\int_{0}^{t}S\left(s\right)\text{d}B_{1}\left(s\right)}{t}=0,\underset{t\to \infty }{\text{lim}}\frac{\int_{0}^{t}E\left(s\right)\text{d}B_{2}\left(s\right)}{t}=0,\underset{t\to \infty }{\text{lim}}\frac{\int_{0}^{t}V_{1}\left(s\right)\text{d}B_{3}\left(s\right)}{t}=0,\\ & \underset{t\to \infty }{\text{lim}}\frac{\int_{0}^{t}V_{2}\left(s\right)\text{d}B_{4}\left(s\right)}{t}=0,\underset{t\to \infty }{\text{lim}}\frac{\int_{0}^{t}A\left(s\right)\text{d}B_{5}\left(s\right)}{t}=0,\underset{t\to \infty }{\text{lim}}\frac{\int_{0}^{t}I\left(s\right)\text{d}B_{6}\left(s\right)}{t}=0,\\ & \underset{t\to \infty }{\text{lim}}\frac{\int_{0}^{t}R\left(s\right)\text{d}B_{7}\left(s\right)}{t}=0, \end{array} \end{array}$$ (17)

*as well as*

$$\begin{array}{c} \begin{array}{cc} & \underset{t\to \infty }{\text{lim}}\frac{\int_{0}^{t}\int_{Y}D_{1}\left(u\right)S\left(s\right)\tilde{N}\left(\text{d}s,\text{d}u\right)}{t}=0,\underset{t\to \infty }{\text{lim}}\frac{\int_{0}^{t}\int_{Y}D_{2}\left(u\right)E\left(s\right)\tilde{N}\left(\text{d}s,\text{d}u\right)}{t}=0,\\ & \underset{t\to \infty }{\text{lim}}\frac{\int_{0}^{t}\int_{Y}D_{3}\left(u\right)V_{1}\left(s\right)\tilde{N}\left(\text{d}s,\text{d}u\right)}{t}=0,\underset{t\to \infty }{\text{lim}}\frac{\int_{0}^{t}\int_{Y}D_{4}\left(u\right)V_{2}\left(s\right)\tilde{N}\left(\text{d}s,\text{d}u\right)}{t}=0,\\ & \underset{t\to \infty }{\text{lim}}\frac{\int_{0}^{t}\int_{Y}D_{5}\left(u\right)A\left(s\right)\tilde{N}\left(\text{d}s,\text{d}u\right)}{t}=0,\underset{t\to \infty }{\text{lim}}\frac{\int_{0}^{t}\int_{Y}D_{6}\left(u\right)I\left(s\right)\tilde{N}\left(\text{d}s,\text{d}u\right)}{t}=0,\\ & \underset{t\to \infty }{\text{lim}}\frac{\int_{0}^{t}\int_{Y}D_{7}\left(u\right)R\left(s\right)\tilde{N}\left(\text{d}s,\text{d}u\right)}{t}=0. \end{array} \end{array}$$ (18)

Proof: Define *X*(*t*) = *S*(*t*) + *E*(*t*) + *V*_1_(*t*) + *V*_2_(*t*) + *A*(*t*) + *I*() + *R*(*t*), Let *Y*(*X*) = *X*^*ϵ*^. Applying Itô formula, we can get

$$\begin{array}{c} \begin{array}{cc} \text{d}Y(X)\leq & LY\text{d}t+\epsilon X^{\epsilon -1}[\sigma_{1}S\left(t\right)\text{d}B_{1}\left(t\right)+\sigma_{2}E\left(t\right)\text{d}B_{2}\left(t\right)+\sigma_{3}V_{1}\left(t\right)\text{d}B_{3}\left(t\right)\\ & +\sigma_{4}V_{2}\left(t\right)\text{d}B_{4}\left(t\right)+\sigma_{5}A\left(t\right)\text{d}B_{5}\left(t\right)+\sigma_{6}I\left(t\right)\text{d}B_{6}\left(t\right)+\sigma_{7}R\left(t\right)\text{d}B_{7}\left(t\right)]\\ & +\int_{Y}X^{\epsilon }[{\left(1+D_{1}\left(u\right)\vee D_{2}\left(u\right)\vee D_{3}\left(u\right)\vee D_{4}\left(u\right)\vee D_{5}\left(u\right)\vee D_{6}\left(u\right)\vee D_{7}\left(u\right)\right)}^{\epsilon }\\ & -D_{1}\left(u\right)\wedge D_{2}\left(u\right)\wedge D_{3}\left(u\right)\wedge D_{4}\left(u\right)\wedge D_{5}\left(u\right)\wedge D_{6}\left(u\right)\wedge D_{7}\left(u\right)]\tilde{N}\left(\text{d}t,\text{d}u\right), \end{array} \end{array}$$

where

$$\begin{array}{c} \begin{array}{cc} LY & \leq \epsilon X^{\epsilon -1}\left(\Lambda -\mu_{1}X-\mu_{2}\left(A+I\right)\right)\\ & +\frac{\epsilon (\epsilon -1)}{2}X^{\epsilon -2}\left(\sigma_{1}^{2}S^{2}+\sigma_{2}^{2}E^{2}+\sigma_{3}^{2}V_{1}^{2}+\sigma_{4}^{2}V_{2}^{2}+\sigma_{5}^{2}A^{2}+\sigma_{6}^{2}I^{2}+\sigma_{7}^{2}R^{2}\right)\\ & +\int_{Y}X^{\epsilon }[{\left(1+D_{1}\left(u\right)\vee D_{2}\left(u\right)\vee D_{3}\left(u\right)\vee D_{4}\left(u\right)\vee D_{5}\left(u\right)\vee D_{6}\left(u\right)\vee D_{7}\left(u\right)\right)}^{\epsilon }\\ & -1-D_{1}\left(u\right)\wedge D_{2}\left(u\right)\wedge D_{3}\left(u\right)\wedge D_{4}\left(u\right)\wedge D_{5}\left(u\right)\wedge D_{6}\left(u\right)\wedge D_{7}\left(u\right)]\lambda \left(\text{d}u\right)\\ & \leq \epsilon X^{\epsilon -2}\left[\Lambda X-\left(\mu_{1}-\frac{\epsilon -1}{2}\sigma^{2}-\frac{1}{\epsilon }d\right)X^{2}\right]\\ & \leq \epsilon X^{\epsilon -2}\left(\Lambda X-bX^{2}\right), \end{array} \end{array}$$ (19)

where constants *σ*^2^ and *d* are defined in assumption (B). Choose *ϵ* > 1 such that

$$\begin{array}{c} \begin{array}{c} b=\mu_{1}-\frac{\epsilon -1}{2}\sigma^{2}-\frac{d}{\epsilon }>0. \end{array} \end{array}$$

So

$$\begin{array}{c} \begin{array}{cc} & \text{d}Y(X)\\ & \leq \epsilon X^{\epsilon -2}\left(\Lambda X-bX^{2}\right)+\epsilon X^{\epsilon -1}[\sigma_{1}S\left(t\right)\text{d}B_{1}\left(t\right)+\sigma_{2}E\left(t\right)\text{d}B_{2}\left(t\right)+\sigma_{3}V_{1}\left(t\right)\text{d}B_{3}\left(t\right)\\ & +\sigma_{4}V_{2}\left(t\right)\text{d}B_{4}\left(t\right)+\sigma_{5}A\left(t\right)\text{d}B_{5}\left(t\right)+\sigma_{6}I\left(t\right)\text{d}B_{6}\left(t\right)+\sigma_{7}R\left(t\right)\text{d}B_{7}\left(t\right)]\\ & +\int_{Y}X^{\epsilon }[{\left(1+D_{1}\left(u\right)\vee D_{2}\left(u\right)\vee D_{3}\left(u\right)\vee D_{4}\left(u\right)\vee D_{5}\left(u\right)\vee D_{6}\left(u\right)\vee D_{7}\left(u\right)\right)}^{\epsilon }\\ & -D_{1}\left(u\right)\wedge D_{2}\left(u\right)\wedge D_{3}\left(u\right)\wedge D_{4}\left(u\right)\wedge D_{5}\left(u\right)\wedge D_{6}\left(u\right)\wedge D_{7}\left(u\right)]\tilde{N}\left(\text{d}t,\text{d}u\right). \end{array} \end{array}$$ (20)

For 0 < *k* < *bϵ*, we have

$$\begin{array}{c} \begin{array}{cc} & \text{d}e^{kt}Y\left(X\left(t\right)\right)\\ & \leq L\left[e^{kt}Y\left(X\left(t\right)\right)\right]\text{d}t+e^{kt}\epsilon X^{\epsilon -1}[\sigma_{1}S\left(t\right)\text{d}B_{1}\left(t\right)+\sigma_{2}E\left(t\right)\text{d}B_{2}\left(t\right)+\sigma_{3}V_{1}\left(t\right)\text{d}B_{3}\left(t\right)\\ & +\sigma_{4}V_{2}\left(t\right)\text{d}B_{4}\left(t\right)+\sigma_{5}A\left(t\right)\text{d}B_{5}\left(t\right)+\sigma_{6}I\left(t\right)\text{d}B_{6}\left(t\right)+\sigma_{7}R\left(t\right)\text{d}B_{7}\left(t\right)]\\ & +e^{kt}\int_{Y}X^{\epsilon }[{\left(1+D_{1}\left(u\right)\vee D_{2}\left(u\right)\vee D_{3}\left(u\right)\vee D_{4}\left(u\right)\vee D_{5}\left(u\right)\vee D_{6}\left(u\right)\vee D_{7}\left(u\right)\right)}^{\epsilon }\\ & -D_{1}\left(u\right)\wedge D_{2}\left(u\right)\wedge D_{3}\left(u\right)\wedge D_{4}\left(u\right)\wedge D_{5}\left(u\right)\wedge D_{6}\left(u\right)\wedge D_{7}\left(u\right)]\tilde{N}\left(\text{d}t,\text{d}u\right). \end{array} \end{array}$$ (21)

Integrating both sides of Eq (21) from 0 to *t* yields

$$\begin{array}{c} \begin{array}{cc} & \int_{0}^{t}\text{d}e^{kt}Y\left(X\left(s\right)\right)\\ & \leq \int_{0}^{t}\left[ke^{ks}Y\left(X\left(s\right)\right)+e^{ks}LY\left(X\left(s\right)\right)\right]\text{d}s\\ & +\int_{0}^{t}e^{ks}\epsilon X^{\epsilon -1}[\sigma_{1}S\left(s\right)\text{d}B_{1}\left(s\right)+\sigma_{2}E\left(s\right)\text{d}B_{2}\left(s\right)+\sigma_{3}V_{1}\left(s\right)\text{d}B_{3}\left(s\right)\\ & +\sigma_{4}V_{2}\left(s\right)\text{d}B_{4}\left(s\right)+\sigma_{5}A\left(s\right)\text{d}B_{5}\left(s\right)+\sigma_{6}I\left(s\right)\text{d}B_{6}\left(s\right)+\sigma_{7}R\left(s\right)\text{d}B_{7}\left(s\right)]\\ & +\int_{0}^{t}e^{ks}\int_{Y}X^{\epsilon }[(1+D_{1}\left(u\right)\vee D_{2}\left(u\right)\vee D_{3}\left(u\right)\vee D_{4}\left(u\right)\vee D_{5}\left(u\right)\\ \;\; & \vee D_{6}\left(u\right)\vee D_{7}{\left(u\right))}^{\epsilon }-D_{1}\left(u\right)\wedge D_{2}\left(u\right)\wedge D_{3}\left(u\right)\wedge D_{4}\left(u\right)\wedge D_{5}\left(u\right)\\ \;\; & \wedge D_{6}\left(u\right)\wedge D_{7}\left(u\right)]\tilde{N}\left(\text{d}t,\text{d}u\right). \end{array} \end{array}$$ (22)

Taking expectation on both sides of Eq (22) yields

$$\begin{array}{c} \begin{array}{c} E\left[e^{kt}Y\left(X\left(t\right)\right)\right]\leq Y\left(X\left(0\right)\right)+E\left[\int_{0}^{t}\left[ke^{ks}Y\left(X\left(s\right)\right)+e^{ks}LY\left(X\left(s\right)\right)\right]\text{d}s\right]. \end{array} \end{array}$$ (23)

Combining Eq (19), we get

$$\begin{array}{c} \begin{array}{cc} ke^{kt}Y\left(X\left(t\right)\right)+e^{ks}LY\left(X\left(t\right)\right)\leq & ke^{kt}X^{\epsilon }\left(t\right)+\epsilon e^{kt}X^{\epsilon -2}\left(t\right)\left[\Lambda X\left(t\right)-bX^{2}\left(t\right)\right]\\ = & \epsilon e^{kt}X^{\epsilon -2}\left(t\right)\left[-\left(b-\frac{k}{\epsilon }\right)X^{2}\left(t\right)+\Lambda X\left(t\right)\right]\\ \leq & \epsilon e^{kt}H, \end{array} \end{array}$$

where

$$\begin{array}{c} \begin{array}{c} H=\underset{X\in R_{+}}{\text{sup}}X^{\epsilon -2}\left[-\left(b-\frac{k}{\epsilon }\right)X^{2}\left(t\right)+\Lambda X\left(t\right)\right]+1. \end{array} \end{array}$$

Thus, it follows from Eq (23) that

$$\begin{array}{c} \begin{array}{cc} E[e^{kt}Y\left(X\left(t\right)\right)]\leq & Y\left(X\left(0\right)\right)+E\left[\int_{0}^{t}\epsilon e^{ks}H\text{d}s\right]\\ \leq & Y\left(X\left(0\right)\right)+\frac{\epsilon H}{k}e^{kt}, \end{array} \end{array}$$

that is, we obtain

$$\begin{array}{c} \begin{array}{c} E\left(X^{\epsilon }\right)\leq \frac{X^{\epsilon }\left(0\right)}{e^{kt}}+\frac{\epsilon H}{k}\leq X^{\epsilon }\left(0\right)+\epsilon H. \end{array} \end{array}$$

For convenience, we denote *Q* = *X*^*ϵ*^(0) + *ϵH*, then we have

$$\begin{array}{c} \begin{array}{c} E(X^{\epsilon }\left(t\right))\leq Q,\;t\geq 0. \end{array} \end{array}$$ (24)

Integrating both sides of Eq (20) from 0 to *t*, for sufficiently small *δ* > 0, *k* = 1, 2, ⋯, we obtain

$$\begin{array}{c} \begin{array}{cc} E[\underset{k\delta <t<(k+1)\delta }{\text{sup}}X^{\epsilon }\left(t\right)]\leq & E{\left(X\left(k\delta \right)\right)}^{\epsilon }+I_{1}+I_{2}\\ \leq & Q+I_{1}+I_{2}, \end{array} \end{array}$$

where

$$\begin{array}{c} \begin{array}{cc} I_{1}= & E\{\underset{k\delta <t<(k+1)\delta }{\text{sup}}|\int_{k\delta }^{t}\epsilon X^{\epsilon -2}\left(s\right)\left[-bX^{2}\left(s\right)+\Lambda X\left(s\right)\right]\text{d}s|\}\\ \leq & l_{1}E\left[\underset{k\delta <t<(k+1)\delta }{\text{sup}}|\int_{k\delta }^{t}X^{\epsilon }\left(s\right)\text{d}s|\right]\\ \leq & l_{1}E\left[\int_{k\delta }^{(k+1)\delta }X^{\epsilon }\left(s\right)\text{d}s\right]\\ \leq & l_{1}\delta E\left[\underset{k\delta <t<(k+1)\delta }{\text{sup}}X^{\epsilon }\left(t\right)\right], \end{array} \end{array}$$

and

$$\begin{array}{c} \begin{array}{cc} I_{2}= & E\{\underset{k\delta <t<(k+1)\delta }{\text{sup}}|\int_{k\delta }^{t}\epsilon X^{\epsilon -1}\left(s\right)[\sigma_{1}S\left(s\right)\text{d}B_{1}\left(s\right)+\sigma_{2}E\left(s\right)\text{d}B_{2}\left(s\right)+\sigma_{3}V_{1}\left(s\right)\text{d}B_{3}\left(s\right)\\ & +\sigma_{4}V_{2}\left(s\right)\text{d}B_{4}\left(s\right)+\sigma_{5}A\left(s\right)\text{d}B_{5}\left(s\right)+\sigma_{6}I\left(s\right)\text{d}B_{6}\left(s\right)+\sigma_{7}R\left(s\right)\text{d}B_{7}\left(s\right)]\\ & +\int_{k\delta }^{t}\int_{Y}X^{\epsilon }[{\left(1+D_{1}\left(u\right)\vee D_{2}\left(u\right)\vee D_{3}\left(u\right)\vee D_{4}\left(u\right)\vee D_{5}\left(u\right)\vee D_{6}\left(u\right)\vee D_{7}\left(u\right)\right)}^{\epsilon }\\ & -D_{1}\left(u\right)\wedge D_{2}\left(u\right)\wedge D_{3}\left(u\right)\wedge D_{4}\left(u\right)\wedge D_{5}\left(u\right)\wedge D_{6}\left(u\right)\wedge D_{7}\left(u\right)]\tilde{N}\left(\text{d}s,\text{d}u\right)|\} \end{array} \end{array}$$

$$\begin{array}{c} \begin{array}{cc} \leq & C_{\epsilon }E{\left[\int_{k\delta }^{(k+1)\delta }\epsilon^{2}X^{2(\epsilon -1)}\left(\sigma_{1}^{2}S^{2}+\sigma_{2}^{2}E^{2}+\sigma_{3}^{2}V_{1}^{2}+\sigma_{4}^{2}V_{2}^{2}+\sigma_{5}^{2}A^{2}+\sigma_{6}^{2}I^{2}+\sigma_{7}^{2}R^{2}\right)\text{d}s\right]}^{\frac{1}{2}}\\ & +C_{\epsilon }E\{\int_{k\delta }^{(k+1)\delta }X^{2\epsilon }\int_{Y}[(1+D_{1}\left(u\right)\vee D_{2}\left(u\right)\vee D_{3}\left(u\right)\vee D_{4}\left(u\right)\vee D_{5}\left(u\right)\\ & \vee D_{6}\left(u\right)\vee D_{7}{\left(u\right))}^{\epsilon }-D_{1}\left(u\right)\wedge D_{2}\left(u\right)\wedge D_{3}\left(u\right)\wedge D_{4}\left(u\right)\wedge D_{5}\left(u\right)\\ & \wedge D_{6}\left(u\right)\wedge D_{7}\left(u\right){{]}^{2}\lambda \left(\text{d}u\right)\text{d}s\}}^{\frac{1}{2}}\\ \leq & C_{\epsilon }\delta^{\frac{1}{2}}[\epsilon \delta +\int_{Y}[(1+D_{1}\left(u\right)\vee D_{2}\left(u\right)\vee D_{3}\left(u\right)\vee D_{4}\left(u\right)\vee D_{5}\left(u\right)\\ & \vee D_{6}\left(u\right)\vee D_{7}{\left(u\right))}^{\epsilon }-D_{1}\left(u\right)\wedge D_{2}\left(u\right)\wedge D_{3}\left(u\right)\wedge D_{4}\left(u\right)\wedge D_{5}\left(u\right)\\ & \wedge D_{6}\left(u\right)\wedge D_{7}\left(u\right){]}^{2}\lambda \left(\text{d}u\right)]E\left[\underset{k\delta <t<(k+1)\delta }{\text{sup}}X^{\epsilon }\right], \end{array} \end{array}$$

where we have used the Burkholder-Davis-Gundy inequality in the aboved, *l*_1_ = *bϵ* and $C_{\epsilon }={\left[\epsilon^{\epsilon +1}/2{\left(\epsilon -1\right)}^{\epsilon -1}\right]}^{\frac{\epsilon }{2}}$ is positive constant. Therefore

$$\begin{array}{c} \begin{array}{cc} & E[\underset{k\delta <t<(k+1)\delta }{\text{sup}}X^{\epsilon }\left(t\right)]\\ \leq & E{\left(X\left(k\delta \right)\right)}^{\epsilon }+[l_{1}\delta +C_{\epsilon }\delta^{\frac{1}{2}}(\epsilon \delta +\int_{Y}[(1+D_{1}\left(u\right)\vee D_{2}\left(u\right)\vee D_{3}\left(u\right)\vee D_{4}\left(u\right)\\ & \vee D_{5}\left(u\right)\vee D_{6}\left(u\right)\vee D_{7}{\left(u\right))}^{\epsilon }-D_{1}\left(u\right)\wedge D_{2}\left(u\right)\wedge D_{3}\left(u\right)\wedge D_{4}\left(u\right)\wedge D_{5}\left(u\right)\\ & \wedge D_{6}\left(u\right)\wedge D_{7}{\left(u\right)]}^{2}\lambda \left(\text{d}u\right))]E\left[\underset{k\delta <t<(k+1)\delta }{\text{sup}}X^{\epsilon }\left(t\right)\right]. \end{array} \end{array}$$

In particular, choose *δ* > 0 such that

$$\begin{array}{c} \begin{array}{cc} & l_{1}\delta +C_{\epsilon }\delta^{\frac{1}{2}}(\epsilon \delta +\int_{Y}[{\left(1+D_{1}\left(u\right)\vee D_{2}\left(u\right)\vee D_{3}\left(u\right)\vee D_{4}\left(u\right)\vee D_{5}\left(u\right)\vee D_{6}\left(u\right)\vee D_{7}\left(u\right)\right)}^{\epsilon }\\ & -D_{1}\left(u\right)\wedge D_{2}\left(u\right)\wedge D_{3}\left(u\right)\wedge D_{4}\left(u\right)\wedge D_{5}\left(u\right)\wedge D_{6}\left(u\right)\wedge D_{7}\left(u\right){]}^{2}\lambda \left(\text{d}u\right))\\ \leq & \frac{1}{2}, \end{array} \end{array}$$

then combine Eq (24), we get

$$\begin{array}{c} \begin{array}{c} E\left[\underset{k\delta <t<(k+1)\delta }{\text{sup}}X^{\epsilon }\left(t\right)\right]\leq 2E{\left(X\left(k\delta \right)\right)}^{\epsilon }\leq 2Q. \end{array} \end{array}$$

Let *ϵ*_*X*_ > 0 be arbitrary. Applying Chebyshev’s inequality, we obtain

$$\begin{array}{c} \begin{array}{cc} P\{\underset{k\delta <t<(k+1)\delta }{\text{sup}}X^{\epsilon }\left(t\right)>\left(k\delta^{1+\epsilon_{X}}\right)\leq & \frac{E[{\text{sup}}_{k\delta <t<(k+1)\delta }X^{\epsilon }\left(t\right)]}{{\left(k\delta \right)}^{1+\epsilon_{X}}}\\ \leq & \frac{2Q}{{\left(k\delta \right)}^{1+\epsilon_{X}}},\;k=1,2,\cdots . \end{array} \end{array}$$

According to Borel-Cantelli lemma, we obtain that for almost all *ω* ∈ Ω

$$\begin{array}{c} \begin{array}{c} \underset{k\delta <t<(k+1)\delta }{\text{sup}}X^{\epsilon }\left(t\right)\leq {\left(k\delta \right)}^{1+\epsilon_{X}} \end{array} \end{array}$$ (25)

holds for all but finitely many *k*. Then, there exists a *k*_0_(*ω*), for almost all *ω* ∈ Ω, for which Eq (25) holds whenever *k* ≥ *k*_0_. Consequently, for almost all *ω* ∈ Ω, if *k* ≥ *k*_0_ and *kδ* < *t* < (*k* + 1)*δ*,

$$\begin{array}{c} \begin{array}{c} \frac{\text{ln}\;X^{\epsilon }\left(t\right)}{\text{ln}\;t}\leq \frac{\left(1+\epsilon_{X}\right)\text{ln}\;\left(k\delta \right)}{\text{ln}\;(k\delta )}=1+\epsilon_{X}. \end{array} \end{array}$$

Hence

$$\begin{array}{c} \begin{array}{c} \underset{t\to \infty }{\text{lim}}\text{sup}\frac{\text{ln}\;X^{\epsilon }\left(t\right)}{\text{ln}\;t}\leq 1+\epsilon_{X}\;\;a.s.. \end{array} \end{array}$$

Let *ϵ*_*X*_ → 0, we obtain

$$\begin{array}{c} \begin{array}{c} \underset{t\to \infty }{\text{lim}}\text{sup}\frac{\text{ln}\;X^{\epsilon }\left(t\right)}{\text{ln}\;t}\leq 1\;\;a.s.. \end{array} \end{array}$$

For $1<\epsilon <1+\frac{2(\mu_{1}-d)}{\sigma^{2}}$, we get $\mu_{1}>\frac{\epsilon -1}{2}\sigma^{2}+d$, and so

$$\begin{array}{c} \begin{array}{c} \underset{t\to \infty }{\text{lim}}\text{sup}\frac{\text{ln}\;X(t)}{\text{ln}\;t}\leq \frac{1}{\epsilon }\;\;a.s.. \end{array} \end{array}$$

Namely, for any small $0<\xi <1-\frac{1}{\epsilon }$, there exists a constant *T* = *T*(*ω*) such that for *t* ≥ *T*,

$$\begin{array}{c} \begin{array}{c} \text{ln}\;X\left(t\right)\leq \left(\frac{1}{\epsilon }+\xi \right)\text{ln}\;t. \end{array} \end{array}$$

So

$$\begin{array}{c} \begin{array}{c} \underset{t\to \infty }{\text{lim}}\text{sup}\frac{X(t)}{t}\leq \frac{t^{\frac{1}{\epsilon }+\xi }}{t}, \end{array} \end{array}$$

which together with the positivity of the solution implies

$$\begin{array}{c} \begin{array}{cc} & \underset{t\to \infty }{\text{lim}}\frac{X(t)}{t}\\ = & \underset{t\to \infty }{\text{lim}}\frac{S\left(t\right)+E\left(t\right)+V_{1}\left(t\right)+V_{2}\left(t\right)+A\left(t\right)+I\left(t\right)+R\left(t\right)}{t}=0,\;\;a.s.. \end{array} \end{array}$$

Then we have

$$\begin{array}{c} \begin{array}{cc} \underset{t\to \infty }{\text{lim}}\frac{S(t)}{t} & =0,\underset{t\to \infty }{\text{lim}}\frac{E(t)}{t}=0,\underset{t\to \infty }{\text{lim}}\frac{V_{1}\left(t\right)}{t}=0,\underset{t\to \infty }{\text{lim}}\frac{V_{2}\left(t\right)}{t}=0,\\ \underset{t\to \infty }{\text{lim}}\frac{A(t)}{t} & =0,\underset{t\to \infty }{\text{lim}}\frac{I(t)}{t}=0,\underset{t\to \infty }{\text{lim}}\frac{R(t)}{t}=0,\;\;a.s.. \end{array} \end{array}$$

By the same way, our subsequent proof is similar to the proof of [38], therefore the lemma has been proven.

In order to get the conditions of diseases extinction, we have

**Theorem 5.1** *If assumption* (*A*),(*B*),(*C*) *hold, let* (*S*(*t*), *E*(*t*), *V*_1_(*t*), *V*_2_(*t*), *A*(*t*), *I*(*t*), $R\left(t\right))\in R_{+}^{7}$ *be a positive solution for* Eq (2), *the initial solution of* Eq (2) *is* (*S*(0), *E*(0), *V*_1_(0), *V*_2_(0), *A*(0), *I*(0), *R*(0)).

*If random threshold*

$$\begin{array}{c} \begin{array}{cc} R_{1}= & \frac{\sigma \Lambda }{\mu_{1}\left(\mu_{1}+\mu_{2}\right)}-\frac{\sigma_{5}^{2}\wedge \sigma_{6}^{2}}{4(\mu_{1}+\mu_{2})}-\frac{\int_{Y}\left(\text{ln}\left(1+D_{5}\left(u\right)\vee D_{6}\left(u\right)\right)-D_{5}\left(u\right)\wedge D_{6}\left(u\right)\right)\lambda \left(\text{d}u\right)}{\mu_{1}+\mu_{2}}<1, \end{array} \end{array}$$

*then the solution of the system has the following property, i.e*

$$\begin{array}{c} \underset{t\to \infty }{\text{lim}}\text{sup}\frac{\text{ln}(A(t)+I(t))}{t}<0. \end{array}$$ (26)

Proof: First, integrating Eq (2) to obtain

$$\begin{array}{c} \begin{array}{cc} \text{d} & \left(S\left(t\right)+E\left(t\right)+V_{1}\left(t\right)+V_{2}\left(t\right)+A\left(t\right)+I\left(t\right)+R\left(t\right)\right)\\ = & [\Lambda -\mu_{1}\left(S\left(t\right)+E\left(t\right)+V_{1}\left(t\right)+V_{2}\left(t\right)+R\left(t\right)\right)-\left(\mu_{1}+\mu_{2}\right)\left(A\left(t\right)+I\left(t\right)\right)]\text{d}t\\ & +\sigma_{1}S\text{d}B_{1}\left(t\right)+\sigma_{2}E\text{d}B_{2}\left(t\right)+\sigma_{3}V_{1}\text{d}B_{3}\left(t\right)+\sigma_{4}V_{2}\text{d}B_{4}\left(t\right)+\sigma_{5}A\text{d}B_{5}\left(t\right)\\ & +\sigma_{6}I\text{d}B_{6}\left(t\right)+\sigma_{7}R\text{d}B_{7}\left(t\right)+a\int_{Y}\left[D_{1}\left(u\right)S\right]\tilde{N}\left(\text{d}t,\text{d}u\right)\\ & +\int_{Y}\left[D_{2}\left(u\right)E\right]\tilde{N}\left(\text{d}t,\text{d}u\right)+\int_{Y}\left[D_{3}\left(u\right)V_{1}\right]\tilde{N}\left(\text{d}t,\text{d}u\right)\\ & +\int_{Y}\left[D_{4}\left(u\right)V_{2}\right]\tilde{N}\left(\text{d}t,\text{d}u\right)+\int_{Y}\left[D_{5}\left(u\right)A\right]\tilde{N}\left(\text{d}t,\text{d}u\right)\\ & +\int_{Y}\left[D_{6}\left(u\right)I\right]\tilde{N}\left(\text{d}t,\text{d}u\right)+\int_{Y}\left[D_{7}\left(u\right)R\right]\tilde{N}\left(\text{d}t,\text{d}u\right). \end{array} \end{array}$$ (27)

Integrating both ends of Eq (27) from 0 to t divides by t, we can obtain

$$\begin{array}{c} \begin{array}{cc} & \frac{S(t)-S(0)}{t}+\frac{E(t)-E(0)}{t}+\frac{V_{1}\left(t\right)-V_{1}\left(0\right)}{t}+\frac{V_{2}\left(t\right)-V_{2}\left(0\right)}{t}\\ & +\frac{A(t)-A(0)}{t}+\frac{I(t)-I(0)}{t}+\frac{R(t)-R(0)}{t}\\ = & A-\mu_{1}\left[\left\langle S\left(t\right)\right\rangle +\left\langle E\left(t\right)\right\rangle +\left\langle V_{1}\left(t\right)\right\rangle +\left\langle V_{2}\left(t\right)\right\rangle +\left\langle R\left(t\right)\right\rangle \right]\\ & -(\mu_{1}+\mu_{2})\left[\left\langle A\left(t\right)\right\rangle +\left\langle I\left(t\right)\right\rangle \right]+\frac{\sigma_{1}}{t}\int_{0}^{t}S\left(s\right)\text{d}B_{1}\left(s\right)\\ & +\frac{\sigma_{2}}{t}\int_{0}^{t}E\left(s\right)\text{d}B_{2}\left(s\right)+\frac{\sigma_{3}}{t}\int_{0}^{t}V_{1}\left(s\right)\text{d}B_{3}\left(s\right)\\ & +\frac{\sigma_{4}}{t}\int_{0}^{t}V_{2}\left(s\right)\text{d}B_{4}\left(s\right)+\frac{\sigma_{5}}{t}\int_{0}^{t}A\left(s\right)\text{d}B_{5}\left(s\right)\\ & +\frac{\sigma_{6}}{t}\int_{0}^{t}I\left(s\right)\text{d}B_{6}\left(s\right)+\frac{\sigma_{7}}{t}\int_{0}^{t}R\left(s\right)\text{d}B_{7}\left(s\right)\\ & +\frac{1}{t}\int_{0}^{t}\int_{Y}\left[D_{1}\left(u\right)S\left(s\right)\right]\tilde{N}\left(\text{d}s,\text{d}u\right)+\frac{1}{t}\int_{0}^{t}\int_{Y}\left[D_{2}\left(u\right)E\left(s\right)\right]\tilde{N}\left(\text{d}s,\text{d}u\right)\\ & +\frac{1}{t}\int_{0}^{t}\int_{Y}\left[D_{3}\left(u\right)V_{1}\left(s\right)\right]\tilde{N}\left(\text{d}s,\text{d}u\right)+\frac{1}{t}\int_{0}^{t}\int_{Y}\left[D_{4}\left(u\right)V_{2}\left(s\right)\right]\tilde{N}\left(\text{d}s,\text{d}u\right)\\ & +\frac{1}{t}\int_{0}^{t}\int_{Y}\left[D_{5}\left(u\right)A\left(s\right)\right]\tilde{N}\left(\text{d}s,\text{d}u\right)+\frac{1}{t}\int_{0}^{t}\int_{Y}\left[D_{6}\left(u\right)I\left(s\right)\right]\tilde{N}\left(\text{d}s,\text{d}u\right)\\ & +\frac{1}{t}\int_{0}^{t}\int_{Y}\left[D_{7}\left(u\right)R\left(s\right)\right]\tilde{N}\left(\text{d}s,\text{d}u\right), \end{array} \end{array}$$ (28)

at this time, we have

$$\begin{array}{c} \begin{array}{cc} \langle E(t)\rangle = & \frac{A}{\mu_{1}}-[\left\langle S\left(t\right)\right\rangle +\langle V_{1}\left(t\right)\rangle +\langle V_{2}\left(t\right)\rangle +\left\langle R\left(t\right)\right\rangle ]\\ & -\frac{\left(\mu_{1}+\mu_{2}\right)}{\mu_{1}}\left[\left\langle A\left(t\right)\right\rangle +\left\langle I\left(t\right)\right\rangle \right]+\phi \left(t\right), \end{array} \end{array}$$ (29)

where

$$\begin{array}{c} \begin{array}{cc} \phi (t)= & \frac{1}{\mu_{1}}[\frac{\sigma_{1}}{t}\int_{0}^{t}S\left(s\right)\text{d}B_{1}\left(s\right)+\frac{\sigma_{2}}{t}\int_{0}^{t}E\left(s\right)\text{d}B_{2}\left(s\right)+\frac{\sigma_{3}}{t}\int_{0}^{t}V_{1}\left(s\right)\text{d}B_{3}\left(s\right)\\ & +\frac{\sigma_{4}}{t}\int_{0}^{t}V_{2}\left(s\right)\text{d}B_{4}\left(s\right)+\frac{\sigma_{5}}{t}\int_{0}^{t}A\left(s\right)\text{d}B_{5}\left(s\right)+\frac{\sigma_{6}}{t}\int_{0}^{t}I\left(s\right)\text{d}B_{6}\left(s\right)\\ & +\frac{\sigma_{7}}{t}\int_{0}^{t}R\left(s\right)\text{d}B_{7}\left(s\right)+\frac{1}{t}\int_{0}^{t}\int_{Y}\left[D_{1}\left(u\right)S\left(s\right)\right]\tilde{N}\left(\text{d}s,\text{d}u\right)\\ & +\frac{1}{t}\int_{0}^{t}\int_{Y}\left[D_{2}\left(u\right)E\left(s\right)\right]\tilde{N}\left(\text{d}s,\text{d}u\right)+\frac{1}{t}\int_{0}^{t}\int_{Y}\left[D_{3}\left(u\right)V_{1}\left(s\right)\right]\tilde{N}\left(\text{d}s,\text{d}u\right)\\ & +\frac{1}{t}\int_{0}^{t}\int_{Y}\left[D_{4}\left(u\right)V_{2}\left(s\right)\right]\tilde{N}\left(\text{d}s,\text{d}u\right)+\frac{1}{t}\int_{0}^{t}\int_{Y}\left[D_{5}\left(u\right)A\left(s\right)\right]\tilde{N}\left(\text{d}s,\text{d}u\right)\\ & +\frac{1}{t}\int_{0}^{t}\int_{Y}\left[D_{6}\left(u\right)I\left(s\right)\right]\tilde{N}\left(\text{d}s,\text{d}u\right)+\frac{1}{t}\int_{0}^{t}\int_{Y}\left[D_{7}\left(u\right)R\left(s\right)\right]\tilde{N}\left(\text{d}s,\text{d}u\right)\\ & -\frac{S(t)-S(0)}{t}-\frac{E(t)-E(0)}{t}-\frac{V_{1}\left(t\right)-V_{1}\left(0\right)}{t}-\frac{V_{2}\left(t\right)-V_{2}\left(0\right)}{t}\\ & -\frac{A(t)-A(0)}{t}-\frac{I(t)-I(0)}{t}-\frac{R(t)-R(0)}{t}]. \end{array} \end{array}$$

Using Itô formula and Eq (2), we can get

$$\begin{array}{c} \begin{array}{cc} & \text{d}\;\text{ln}(A(t)+I(t))\\ & =[\frac{1}{A(t)+I(t)}\left(\sigma E-\left(\gamma_{1}+\mu_{1}+\mu_{2}\right)A-\left(\gamma_{2}+\mu_{1}+\mu_{2}\right)I\right)-\frac{\sigma_{5}^{2}A^{2}\left(t\right)+\sigma_{6}^{2}I^{2}\left(t\right)}{2{\left(A\left(t\right)+I\left(t\right)\right)}^{2}}\\ & -\int_{Y}\left(\frac{D_{5}\left(u\right)A\left(t\right)+D_{6}\left(u\right)I\left(t\right)}{A(t)+I(t)}-\text{ln}\frac{\left(1+D_{5}\left(u\right)A\left(t\right)\right)+\left(1+D_{6}\left(u\right)I\left(t\right)\right)}{A(t)+I(t)}\right)\lambda \left(\text{d}u\right)]\text{d}t\\ & +\frac{\sigma_{5}A\left(t\right)}{A(t)+I(t)}\text{d}B_{5}\left(t\right)+\frac{\sigma_{6}I\left(t\right)}{A(t)+I(t)}\text{d}B_{6}\left(t\right)\\ & +\int_{Y}\text{ln}\frac{\left(1+D_{5}\left(u\right)A\left(t\right)\right)+\left(1+D_{6}\left(u\right)I\left(t\right)\right)}{A(t)+I(t)}\tilde{N}\left(\text{d}t,\text{d}u\right)\\ & \leq [\sigma E-\left(\mu_{1}+\mu_{2}\right)-\frac{\left(\sigma_{5}^{2}\wedge \sigma_{6}^{2}\right)\left(A\left(t\right)+I\left(t\right)\right)}{2{\left(A\left(t\right)+I\left(t\right)\right)}^{2}}\\ & -\int_{Y}\left(\text{ln}\left(1+D_{5}\left(u\right)\vee D_{6}\left(u\right)\right)-D_{5}\left(u\right)\wedge D_{6}\left(u\right)\right)\lambda \left(\text{d}u\right)]\text{d}t\\ & +\frac{\sigma_{5}A\left(t\right)}{A(t)+I(t)}\text{d}B_{5}\left(t\right)+\frac{\sigma_{6}I\left(t\right)}{A(t)+I(t)}\text{d}B_{6}\left(t\right)\\ & +\int_{Y}\text{ln}\left(1+D_{5}\left(u\right)\vee D_{6}\left(u\right)\right)\tilde{N}\left(\text{d}t,\text{d}u\right). \end{array} \end{array}$$ (30)

Integrating the Eq (30) from 0 to *t* and divide by *t*, so there is

$$\begin{array}{c} \begin{array}{cc} & \frac{\text{ln}(A(t)+I(t))-\text{ln}(A(0)+I(0))}{t}\\ \leq & \sigma \left\langle E\left(t\right)\right\rangle -\left(\mu_{1}+\mu_{2}\right)-\frac{\left(\sigma_{5}^{2}\wedge \sigma_{6}^{2}\right)}{4}\\ & -\int_{Y}\left(\text{ln}\left(1+D_{5}\left(u\right)\vee D_{6}\left(u\right)\right)-D_{5}\left(u\right)\wedge D_{6}\left(u\right)\right)\lambda \left(\text{d}u\right)\\ & +\frac{1}{t}\int_{0}^{t}\frac{\sigma_{5}A\left(s\right)}{A(s)+I(s)}\text{d}B_{5}\left(s\right)+\frac{1}{t}\int_{0}^{t}\frac{\sigma_{6}I\left(s\right)}{A(s)+I(s)}\text{d}B_{6}\left(s\right)\\ & +\frac{1}{t}\int_{0}^{t}\int_{Y}\text{ln}\left(1+D_{5}\left(u\right)\vee D_{6}\left(u\right)\right)\tilde{N}\left(\text{d}s,\text{d}u\right)\\ \leq & \sigma \left\langle E\left(t\right)\right\rangle -\left(\mu_{1}+\mu_{2}\right)-\frac{\sigma_{5}^{2}\wedge \sigma_{6}^{2}}{4}\\ & -\int_{Y}\left(\text{ln}\left(1+D_{5}\left(u\right)\vee D_{6}\left(u\right)\right)-D_{5}\left(u\right)\wedge D_{6}\left(u\right)\right)\lambda \left(\text{d}u\right)\\ & +\frac{1}{t}\int_{0}^{t}\frac{\sigma_{5}A\left(s\right)}{A(s)+I(s)}\text{d}B_{5}\left(s\right)+\frac{1}{t}\int_{0}^{t}\frac{\sigma_{6}I\left(s\right)}{A(s)+I(s)}\text{d}B_{6}\left(s\right)+\frac{M(t)}{t}, \end{array} \end{array}$$ (31)

where

$$\begin{array}{c} \begin{array}{c} M\left(t\right)=\int_{0}^{t}\int_{Y}\text{ln}\left(1+D_{5}\left(u\right)\vee D_{6}\left(u\right)\right)\tilde{N}\left(\text{d}s,\text{d}u\right). \end{array} \end{array}$$

Using assumption (C), we can obtain

$$\begin{array}{c} \begin{array}{c} \left\langle M,M\right\rangle =t\int_{Y}{\left[\text{ln}\left(1+D_{5}\left(u\right)\vee D_{6}\left(u\right)\right)\right]}^{2}\lambda \left(\text{d}u\right)<tD. \end{array} \end{array}$$

According to the strong number theorem, we have

$$\begin{array}{c} \begin{array}{cc} & \underset{t\to \infty }{\text{lim}}\frac{M(t)}{t}=0,\\ & \underset{t\to \infty }{\text{lim}}\frac{1}{t}\int_{0}^{t}\frac{\sigma_{5}A\left(s\right)}{A(s)+I(s)}\text{d}B_{5}\left(s\right)=0,\\ & \underset{t\to \infty }{\text{lim}}\frac{1}{t}\int_{0}^{t}\frac{\sigma_{6}I\left(s\right)}{A(s)+I(s)}\text{d}B_{6}\left(s\right)=0. \end{array} \end{array}$$ (32)

It can be get by combining Eqs (29), (31) and (32)

$$\begin{array}{c} \begin{array}{cc} & \underset{t\to \infty }{\text{lim}}\text{sup}\frac{\text{ln}(A(t)+I(t))}{t}\\ ⩽ & \frac{\sigma \Lambda }{\mu_{1}}-\left(\mu_{1}+\mu_{2}\right)-\frac{\sigma_{5}^{2}\wedge \sigma_{6}^{2}}{4}\\ & -\int_{Y}\left(\text{ln}\left(1+D_{5}\left(u\right)\vee D_{6}\left(u\right)\right)-D_{5}\left(u\right)\wedge D_{6}\left(u\right)\right)\lambda \left(\text{d}u\right)\\ = & \left(\mu_{1}+\mu_{2}\right)\left(R_{1}-1\right), \end{array} \end{array}$$ (33)

where the random threshold is denoted as

$$\begin{array}{c} \begin{array}{cc} R_{1}= & \frac{\sigma \Lambda }{\mu_{1}\left(\mu_{1}+\mu_{2}\right)}-\frac{\sigma_{5}^{2}\wedge \sigma_{6}^{2}}{4(\mu_{1}+\mu_{2})}-\frac{\int_{Y}\left(\text{ln}\left(1+D_{5}\left(u\right)\vee D_{6}\left(u\right)\right)-D_{5}\left(u\right)\wedge D_{6}\left(u\right)\right)\lambda \left(\text{d}u\right)}{\mu_{1}+\mu_{2}}. \end{array} \end{array}$$

If *R*_1_ < 1, There are

$$\begin{array}{c} \underset{t\to \infty }{\text{lim}}\text{sup}\frac{\text{ln}(A(t)+I(t))}{t}<0. \end{array}$$

Therefore, this conclusion is proved.

According to the random threshold *R*_1_ in the above theorem, if *R*_1_ < 1, the disease will disappear for a period of time, otherwise the disease will continue and further develop into endemic diseases.

## 6 Numerical simulations

In this section, numerical simulations will be conducted to verify the proposed theoretical results. To this end, we will apply the Euler numerical approximation method [39, 40] to calculate Eq (2). By assumption (A) and the constraints on *c*_*i*_ > 0(*i* = 1, ⋯, 5) in Theorem 4.1, we provide the following parameters for numerical simulation:

$$\begin{array}{c} \begin{array}{cc} \Lambda & =0.01,\;\beta_{1}=1.58,\;\beta_{2}=1.95,\;\sigma =0.56,\;\omega =0.2,\alpha =0.19,\\ \gamma_{1}= & 0.65,\;\gamma_{2}=0.83,\;\mu_{1}=0.052,\;\mu_{2}=0.028,\;\rho_{1}=0.92,\;\rho_{2}=0.89,\alpha_{2}=0.35, \end{array} \end{array}$$

at this time, the disease-free equilibrium is *P*_0_ = (0.0103, 0, 0.01, 0.0127, 0, 0, 0.1592).

Based on the constraints proposed in the theorem and the conditions in the hypothesis, we choose

$$\begin{array}{c} \begin{array}{c} \sigma_{1}=0.25,\;\sigma_{2}=0.33,\;\sigma_{3}=0.18,\;\sigma_{4}=0.35,\sigma_{5}=0.35,\;\sigma_{6}=0.42,\;\sigma_{7}=0.23,\\ D_{1}=0.3,\;D_{2}=0.62,\;D_{3}=0.42,\;D_{4}=0.27,\;D_{5}=0.25,\;D_{6}=0.42,\;D_{7}=0.33. \end{array} \end{array}$$

In this case, the random threshold *R*_1_ = 0.0855 < 1.

**Fig 2:**
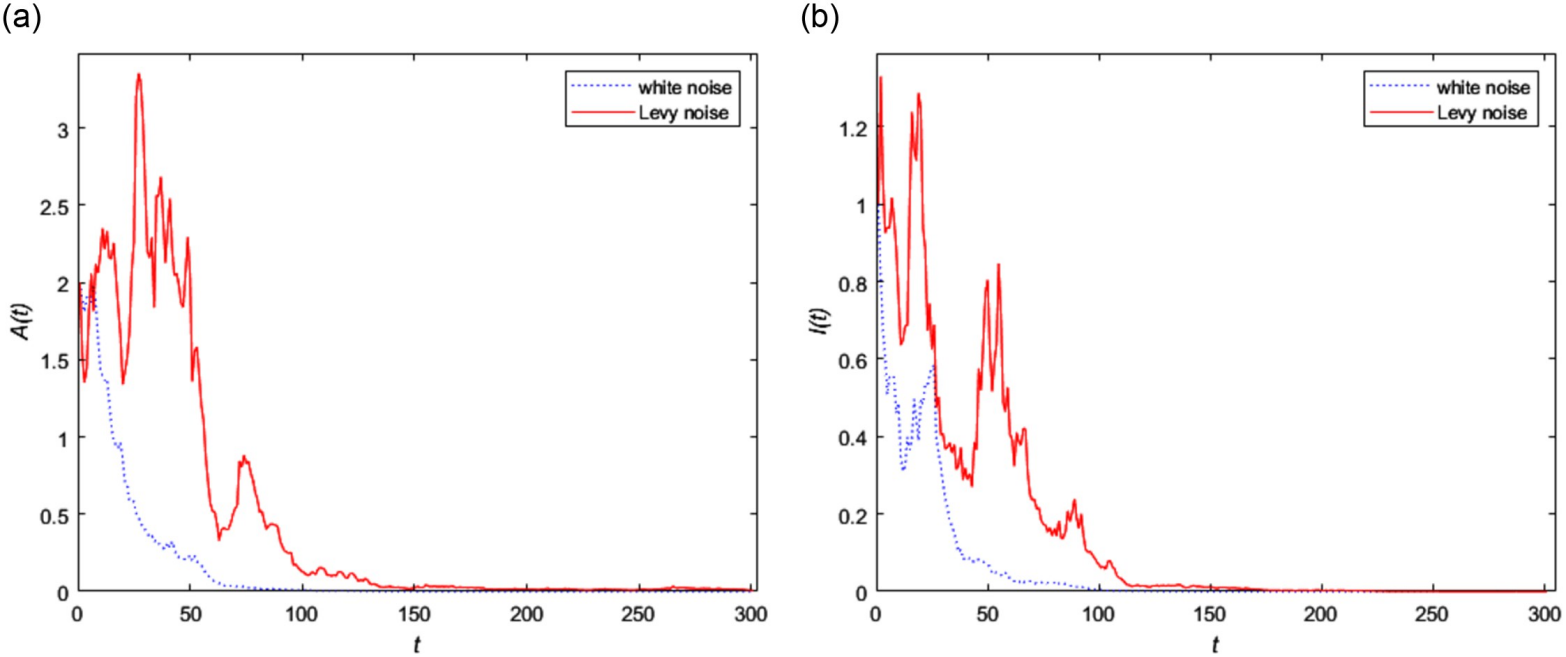
The trend of infected persons in stochastic model (2) with white noise and Lévy noise.

The initial value of Eq (2) is set as (1, 1, 0, 0, 2, 1, 0). Using the data given above, Fig 2 shows the asymptotic behavior of the stochastic model with different noise at the disease-free equilibrium point. From Fig 2, it can be clearly observed that the two curves for *A*(*t*) shown in Fig 2(a) and *I*(*t*) shown in Fig 2(b) with different noise gradually approach zero, which means that the infected persons of the disease gradually approach extinction. That is consistent with the theory we found about the disappearance of infected people. It also can be seen from the Fig 2 that the curve with Lévy noise fluctuates greatly, which indicates that Lévy noise has an prominent effect on both asymptomatic and infected persons. Obviously it has a greater impact on asymptomatic infected people shown in Fig 2(a) for Lévy noise, which means that asymptomatic infected people are not easy to find under common conditions. Therefore the control measures for asymptomatic infected people should be scientifically deployed.

**Fig 3:**
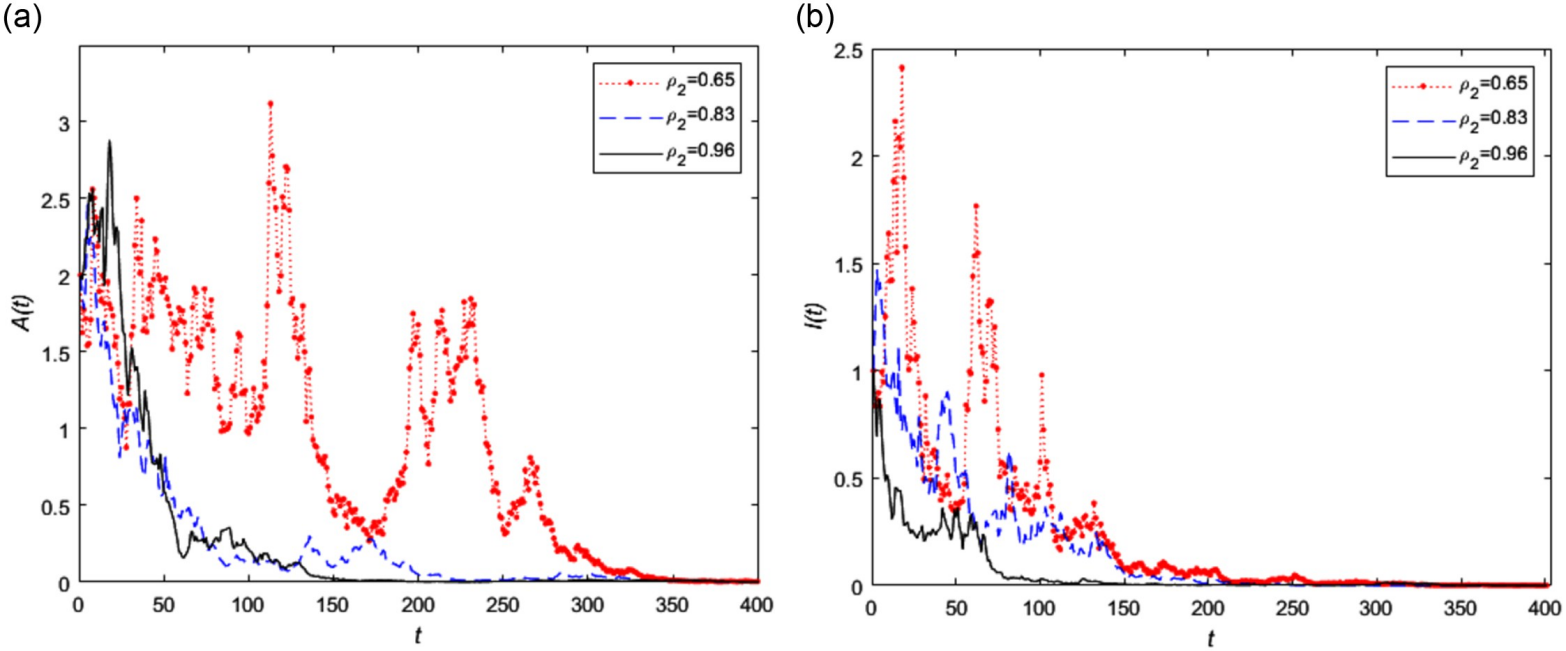
The trend of infected persons in stochastic model (2) with different secondary vaccination rate *ρ*_2_, where *ρ*_2_ = 0.65, 0.83, 0.96, respectively.

Based on the above parameters, we let *ρ*_2_ is equal to 0.65, 0.83 and 0.96 respectively, the extinction trend of asymptomatic infected person *A*(*t*) and symptomatic infected person *I*(*t*) is shown in Fig 3(a) and 3(b) respectively. It can be clearly seen from Fig 3 that with the increase of secondary vaccination rate, the two types of infected populations gradually become extinct at a faster rate, which means that the extinction rate of the disease is faster.

**Fig 4:**
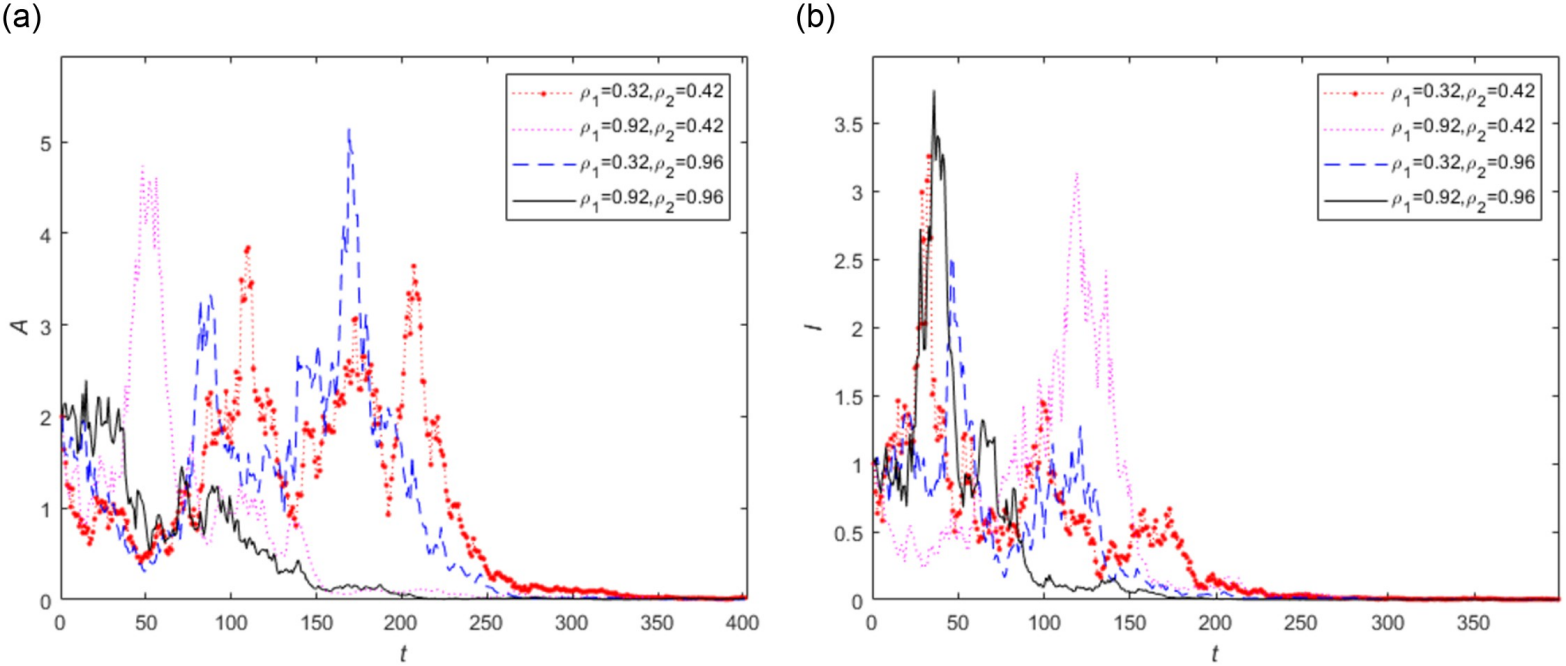
The trend of infected persons in stochastic model (2) with vaccination rate *ρ*_1_ and *ρ*_2_, where (1)*ρ*_1_ = 0.32, *ρ*_2_ = 0.42, (2)*ρ*_1_ = 0.92, *ρ*_2_ = 0.42, (3) *ρ*_1_ = 0.32, *ρ*_2_ = 0.96, (4)*ρ*_1_ = 0.92, *ρ*_2_ = 0.96.

Next, in order to further investigate the impact of vaccination on diseases, the extinction trends of infected individuals are plotted under different vaccination rates in Fig 4. It is observed from Fig 4 that the trend of disease extinction is faster when both vaccination rates are high. However for lower value of vaccination rates, for example *ρ*_1_ = 0.32, *ρ*_2_ = 0.42, the longer it takes to achieve disease extinction. This indicates that increasing the vaccination rate has a significant impact on diseases, and when the vaccination rate is low, prevention and control work will take longer.

In addition, the curve fluctuation of asymptomatic infected persons fluctuates significantly shown in Figs 3(a) and 4(a) when the vaccination rates is smaller, which means that in real life, the population’s immunity decreases after reducing vaccination. Due to the difficulty in detecting asymptomatic infected individuals, there will be significant changes in the number of infected individuals in such situations. Therefore, we should improve the secondary vaccination rate and increase the body immunity rate to effectively control the epidemic in real life.

**Fig 5:**
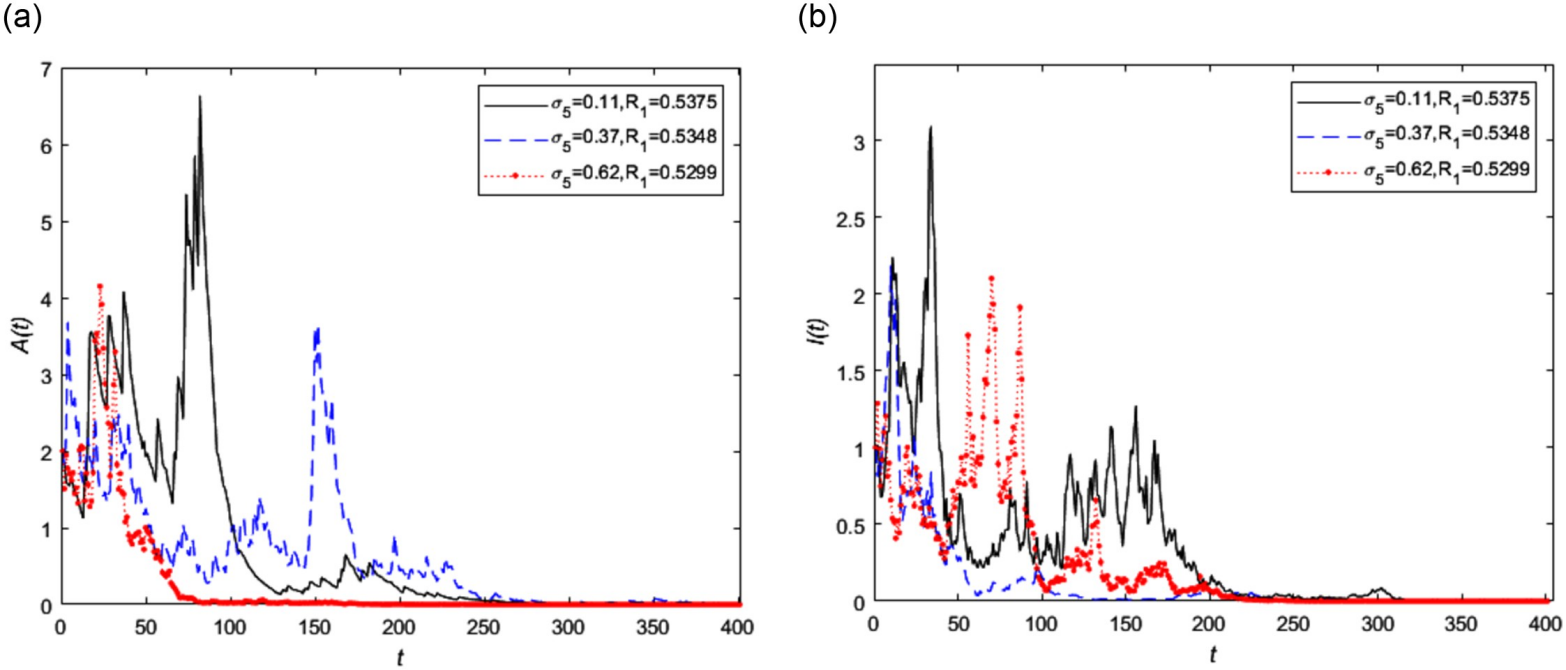
The trend of infected persons in stochastic model (2) with different white noise intensity *σ*_5_, where *σ*_5_ = 0.11, 0.37, 0.62, respectively and *σ*_6_ = 0.62.

According to Theorem 5.1, the random threshold *R*_1_ will affect the extinction of the disease, therefore it is necessary to analyze the influence of *R*_1_ on its extinction. Assume that the given system parameters and partial noise intensity remain unchanged, we make that *σ*_6_ = 0.62 and *σ*_5_ is 0.11, 0.37 and 0.62 respectively, then the random threshold value *R*_1_ is equal to 0.5375, 0.5348 and 0.5299 respectively. From this, we know that the influence of white noise intensity *σ*_5_ on random threshold is insensitive. The trend of asymptomatic infected person *A*(*t*) and symptomatic infected person *I*(*t*) with different white noise intensity *σ*_5_ is shown in Fig 5(a) and 5(b) respectively. It can be intuitively concluded that when the white noise disturbance intensities increase, the fluctuation amplitude of the curve is relatively low, and with the increase of noise disturbance intensity, the extinction rate of the disease will be faster. As can be seen from Fig 5 that the change of asymptomatic infected person more fluctuated significantly shown in Fig 5(a) with the increase of *σ*_5_.

**Fig 6:**
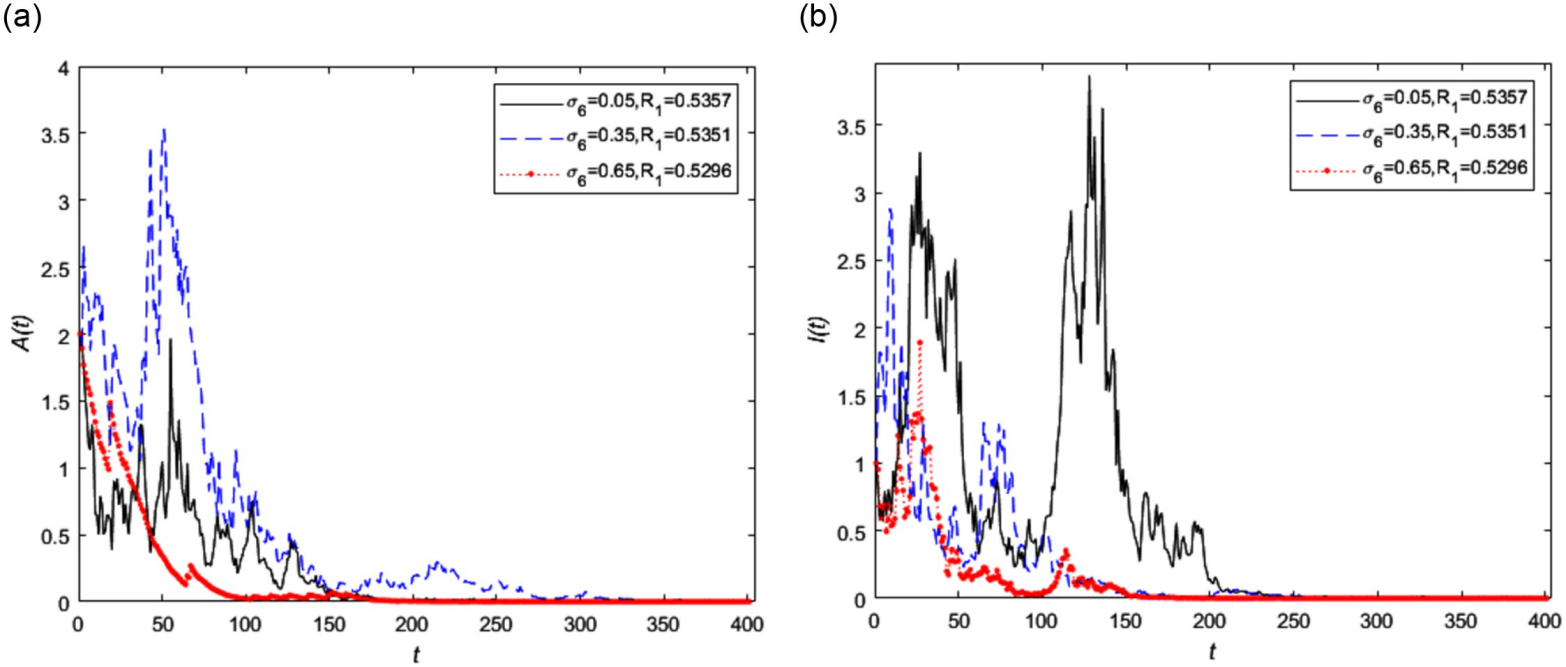
The trend of infected persons in stochastic model (2) with different white noise intensity *σ*_6_, where *σ*_6_ = 0.05, 0.35, 0.65, respectively and *σ*_5_ = 0.65.

In addition, We set *σ*_5_ = 0.65 and *σ*_6_ is equal to 0.05, 0.35 and 0.65 respectively, the other parameters remain unchanged, then the random threshold *R*_1_ is equal to 0.5375, 0.5351 and 0.5296 respectively. It is also found that the the influence of white noise intensity *σ*_6_ on random threshold is insensitive. The trend of asymptomatic infected person *A*(*t*) and symptomatic infected person *I*(*t*) with different white noise intensity *σ*_6_ is shown in Fig 6(a) and 6(b) respectively. It can be seen from Fig 6 that the change of symptomatic infected people fluctuates more obviously shown in Fig 6(b) as *σ*_6_ is increased.

Figs 5 and 6 indicate that the minor events disturbances have insensitive impact on the extinction of disease. However the intensity *σ*_5_ of the white noise *B*_5_(*t*) caused by the environment changes for asymptomatic infected people just affected themselves obviously. The same as the *σ*_6_ to symptomatic infected person. Therefore epidemic prevention and control should be treated in different categories for different kind of infected people.

**Fig 7:**
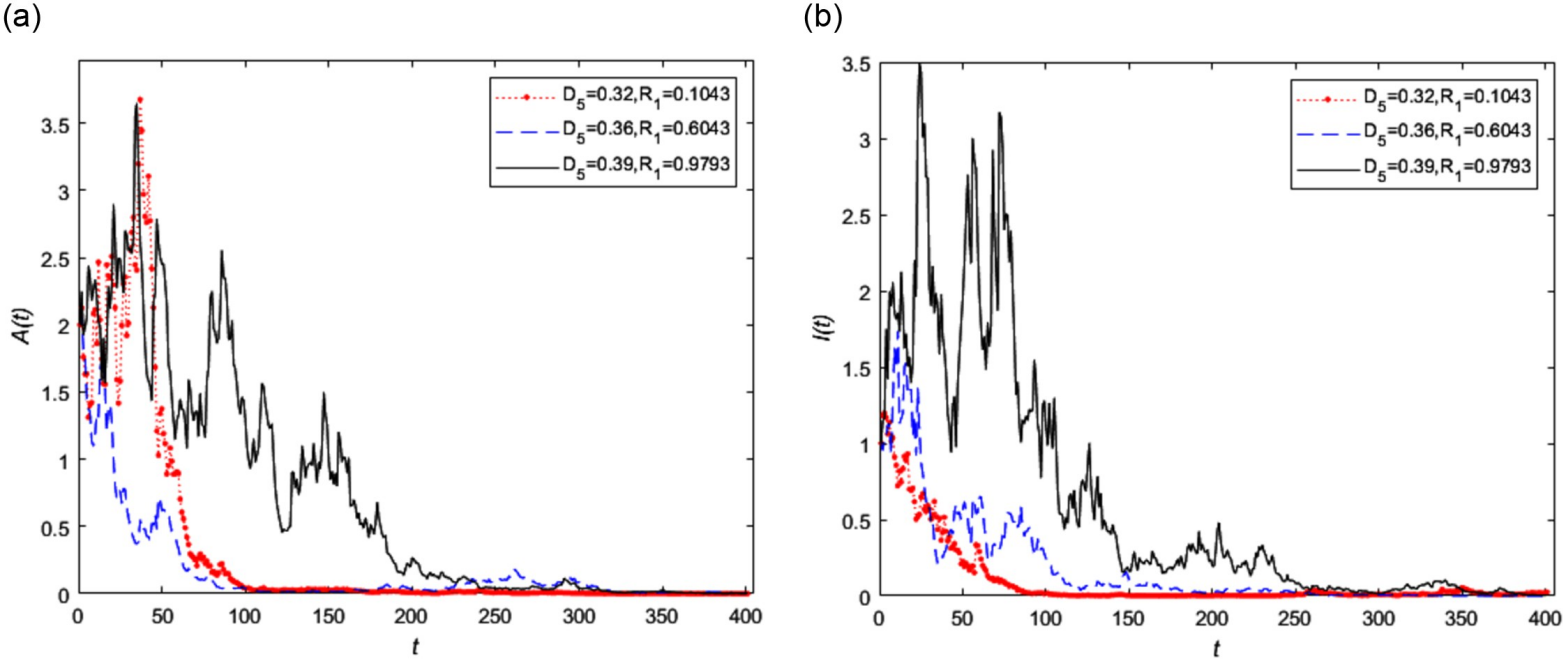
The trend of infected persons in stochastic model (2) with different diffusion coefficient *D*_5_, where *D*_5_ = 0.32, 0.36, 0.39, respectively and *D*_6_ = 0.45.

Let the proposed parameters remain unchanged, *D*_6_ = 0.45 and *D*_5_ is chosen as 0.32, 0.36 and 0.39 respectively, the random threshold *R*_1_ is equal to 0.1043, 0.6043 and 0.9793 respectively. It is obvious that the small change of *D*_5_ can make the lager change of *R*_1_. The trend of asymptomatic infected person *A*(*t*) and symptomatic infected person *I*(*t*) in stochastic model (2) with different diffusion coefficient *D*_5_ is shown in Fig 7(a) and 7(b) respectively. It can be seen from Fig 7 that the later to disappear of asymptomatic infected people shown in Fig 7(a) as *D*_5_ is increased, which means that the sudden fluctuation encountered by asymptomatic infected people have a stronger impact on the extinction of disease.

**Fig 8:**
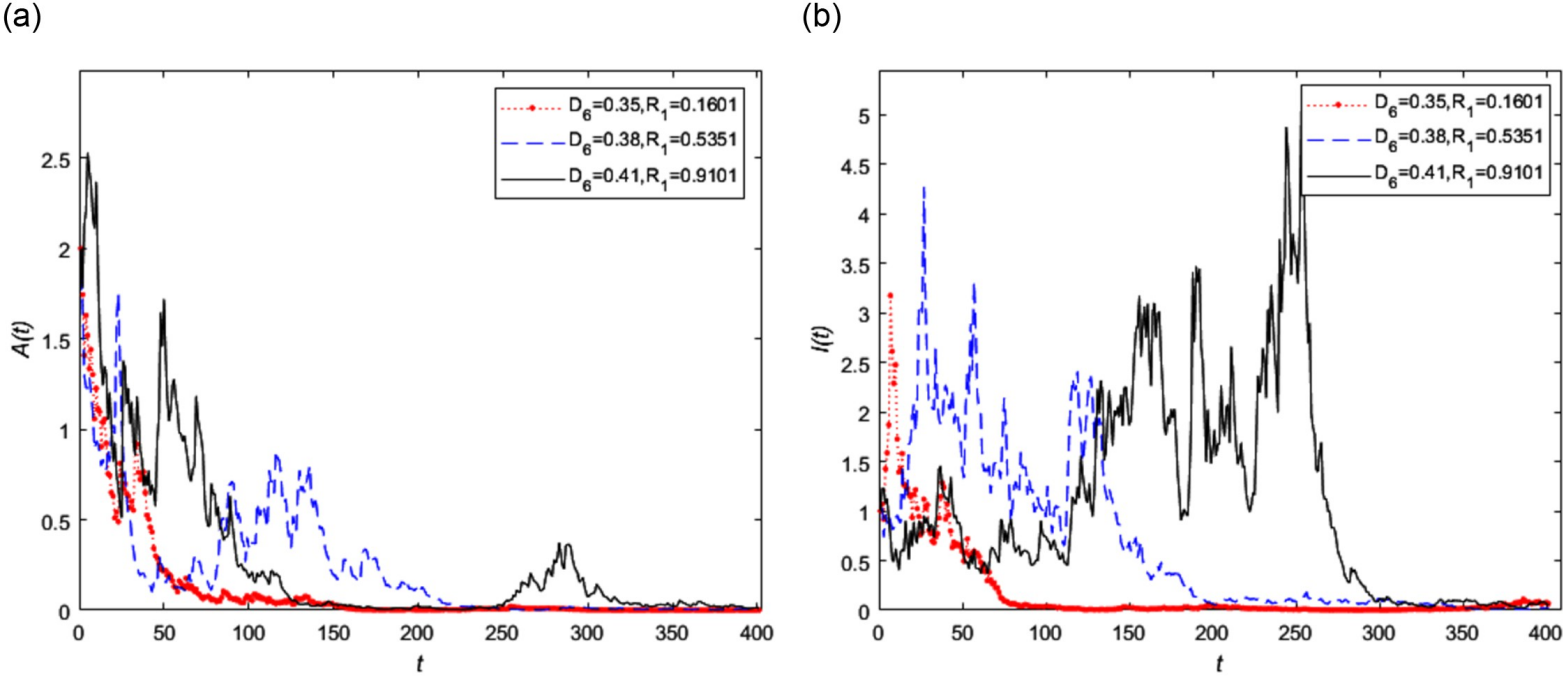
The trend of infected persons in stochastic model (2) with different diffusion coefficient *D*_6_, where *D*_6_ = 0.35, 0.38, 0.41, respectively and *D*_5_ = 0.56.

In addition, let *D*_5_ = 0.56 and *D*_6_ be 0.35, 0.38 and 0.41 respectively, then the random threshold *R*_1_ at this time is 0.1601, 0.5351 and 0.9101 respectively. We can find that the small change of *D*_6_ can make the lager change of *R*_1_. The trend of asymptomatic infected person *A*(*t*) and symptomatic infected person *I*(*t*) with different diffusion coefficient *D*_6_ is shown in Fig 8(a) and 8(b) respectively. It can be seen from Fig 8 that when the diffusion coefficient *D*_6_ of Lévy noise caused by symptomatic infected persons *I*(*t*) changes, the symptomatic infected persons changed significantly shown in Fig 8(b).

From the Figs 7 and 8, we know the smaller the random threshold *R*_1_, the faster the disease reaches the disease-free equilibrium point. This means that both the diffusion coefficients have a sensitive impact on disease extinction. In real life, some emergencies can affect the spread and control of infectious disease. The drug treatment and passive physical isolation play a role in promoting the extinction of the disease. At the same time, the virus detection ability and drug treatment need to be further improved.

## 7 Conclusions

Nowadays, infectious disease models with vaccination have been used by more and more researchers to predict the infectious diseases. On the other hand, in real life, there are many random factors which affect the spread of the epidemic, such as large scale human activities, preventional policies and drug improvement. On this basis, the mathematical epidemic model of vaccination affected by white noise and Lévy noise is established, then the dynamics of the stochastic model was analyzed.

Firstly, the model is proved to have a globally unique positive solution by establishing Lyapunov function. Secondly, the asymptotic behavior of disease-free equilibrium is studied. Then, we obtain the theoretical results about the disease extinction for the random threshold *R*_1_. If *R*_1_ < 1, the disease tends to go extinct. Finally, the numerical simulation results show that the Lévy noise has a great influence on disease dynamics which verified the theoretical proof. When the random threshold of the infectious disease is less than 1, the proposed random model can become extinct in an average sense. The main reason may be random interference, which can lead to the disappearance of diseases due to large white noise interference. However, in the stochastic model driven by Lévy noise, the factors affected by white noise cannot account for the majority of the reasons, and Lévy noise is the main factor determining the development of diseases. Levy noise may also inhibit the spread of diseases or promote their spread.

This work provides a stochastic infectious disease dynamics model with secondary vaccination, which helps to understand the impact of vaccination and random noise on infectious disease prevention and control. In terms of disease prevention and control in the real world, we find that when some sudden situations occur for infected people, it may directly promote or control the spread of infectious diseases. For example, develop more effective vaccines and drugs for infected people and let more people to receive multiple vaccinations, which must provide a powerful help to control the spread of infectious disease. In addition, during the outbreak of the epidemic, susceptible populations can be encouraged to receive vaccines to improve their effectiveness, and efforts should be made to increase the immunity of the population through such measures to prevent the further spread and spread of infectious diseases.

Based on the Virus evolution and technological development, we need improve the researches for the infection disease deeply. Such as considering the effects of impulsive perturbations on system (2) or the impact of viral mutations on vaccine administration. In our future researches the machine learning method, data-driven modeling and intelligent prediction will be applied to explore the infectious disease more effectively.
